# Views of People With Psychosis About Algorithm-Based Relapse Prediction and Data Sharing: Qualitative Study

**DOI:** 10.2196/86753

**Published:** 2026-04-10

**Authors:** Emily Eisner, Hannah Ball, John Ainsworth, Matteo Cella, Richard J Drake, Daniel Elton, Sophie Faulkner, Kathryn Greenwood, Andrew Gumley, Gillian Haddock, Kimberley M Kendall, Alex Kenny, Tor-Ivar Krogsæter, Jane Lees, Shôn Lewis, Alie Phiri, Matthias Schwannauer, Rebecca Turner, Annabel E L Walsh, James Walters, Til Wykes, Uzma Zahid, Sandra Bucci

**Affiliations:** 1 Division of Psychology and Mental Health, School of Health Sciences, Faculty of Biology, Medicine and Health, University of Manchester, Jean McFarlane Building, Manchester, United Kingdom, +44 1613066000; 2 Greater Manchester Mental Health NHS Foundation Trust, Manchester, United Kingdom; 3 Division of Informatics, Imaging and Data Sciences, School of Health Sciences, Faculty of Biology, Medicine and Health, University of Manchester, Manchester, United Kingdom; 4 Department of Psychology, Institute of Psychiatry, Psychology & Neuroscience, King's College London, London, United Kingdom; 5 South London and Maudsley NHS Foundation Trust, London, United Kingdom; 6 McPin Foundation, London, United Kingdom; 7 School of Psychology, University of Sussex, Falmer, United Kingdom; 8 Sussex Partnership NHS Foundation Trust, Hove, United Kingdom; 9 School of Health and Wellbeing, University of Glasgow, Glasgow, United Kingdom; 10 NHS Greater Glasgow and Clyde, Glasgow, United Kingdom; 11 Centre for Neuropsychiatric Genetics and Genomics, Division of Psychological Medicine and Clinical Neurosciences, Cardiff University, Cardiff, United Kingdom; 12 School of Health in Social Science, University of Edinburgh, Edinburgh, United Kingdom; 13 NHS Lothian, Edinburgh, United Kingdom

**Keywords:** psychosis, mobile app, relapse, passive sensing, remote monitoring, wearables, machine learning, prediction, prevention, qualitative, acceptability

## Abstract

**Background:**

Preventing relapses of psychosis is difficult and important. Digital remote monitoring (DRM) systems are being developed and tested to support this. Increasingly, these systems use algorithm-based relapse prediction. Hence, understanding stakeholder views about algorithmic prediction is crucial. Existing qualitative work has explored health professionals’ views, but very few studies have examined the perspectives of people with psychosis on this topic.

**Objective:**

This paper aimed to provide an in-depth examination of the views of people with psychosis regarding algorithmic relapse prediction within a DRM system that incorporates active symptom monitoring and passive sensing data.

**Methods:**

People with psychosis (n=58) were recruited from 6 geographically distinct areas of the United Kingdom. They participated in semistructured qualitative interviews exploring their views about using a DRM system that predicts psychosis relapse based on a machine learning algorithm. Transcripts were analyzed using reflexive thematic analysis. People with lived experience of psychosis were involved extensively in study design, analysis, and reporting.

**Results:**

Findings were described across 4 themes. First, *accuracy* was a prominent theme. Participants emphasized that transparency about algorithm sensitivity and specificity is crucial and discussed the risks of the relapse prediction algorithm producing false positives (flagging that someone was relapsing when they were not) and false negatives (missing actual relapses). In both cases, participants said that errors may be partially mitigated through a *human-in-the-loop* approach (theme 2), with DRM blended with human oversight, from clinicians or a dedicated digital monitoring team, and calibrated based on service user, carer, and clinician feedback. The third theme, *trust, fears, and choice,* noted the interplay between users’ trust in the DRM system and their relationship with the clinical team. This theme described participants’ fears about potential overreactions (hospitalization or excessive medication) or underreactions (no additional support) from the clinical team in response to algorithm-generated relapse predictions. It emphasized the importance of retaining choice around the use of relapse detection algorithms and the sharing of personal data. The final theme described participants’ views about the *benefits of using a relapse prediction algorithm*, including facilitating early intervention, triaging care according to need, minimizing human bias in assessment, and efficiency in saving staff time.

**Conclusions:**

People with psychosis acknowledged potential benefits of algorithm-assisted relapse prediction for receiving timely or efficient care, but with several caveats. Algorithm-generated relapse alerts need to be sufficiently accurate and must be interpreted, with understanding of their limitations, by a trustworthy human who is aware of the relevant context. Algorithm-based relapse predictions should only be used with valid consent, in a way that promotes and respects the autonomy and voice of service users and avoids increasing the use of excessive restriction.

## Introduction

Although approximately half of individuals experiencing a first episode of psychosis reach symptom remission [1], around half experience a relapse within 3 years [2]. Relapses are linked to significant adverse outcomes for individuals, including disrupted educational or occupational trajectories [3,4], reduced functioning [4,5], poorer clinical prognoses [6,7], increased mortality risk [8-10], and considerable financial costs to health services, primarily due to inpatient admissions [11-14]. Standard approaches to community-based mental health monitoring typically depend on individuals recalling symptoms from previous weeks or months during infrequent clinical appointments. Due to recall bias, these retrospective self-reports often lack accuracy, delaying the timely detection of emerging relapse indicators. Given the need for prompt and precise intervention in psychosis, it is important to detect relapses early and intervene swiftly, without further burdening overstretched mental health services or individuals with psychosis.

To meet this need, the multisite CONNECT study involves co-designing and evaluating a digital remote monitoring (DRM) system that uses a machine learning (ML)–generated algorithm to predict relapse. As the algorithm is still under codevelopment, detailed specifications cannot yet be disclosed. The algorithm will integrate information from multiple data streams, including active symptom monitoring (ASM) and passive sensing. ASM uses self-reported smartphone questionnaires to track symptoms [15-17]. Passive sensing uses unobtrusively collected sensor data (eg, GPS and accelerometry) to infer behavioral and social functioning (eg, movement patterns and sleep disturbances) without requiring user input [18-20]. The resulting DRM system will monitor mental health status and alert service users and/or clinicians to early signs of relapse, aiming to facilitate timely intervention and mitigate the potentially severe consequences of a full relapse.

Given the well-recognized challenges of integrating new technologies into health care, digital systems must be developed with implementation considerations at the forefront [21-23]. End users are central to this process; their engagement ultimately determines whether such systems are adopted and maintained [24]. Therefore, understanding the perspectives of people with psychosis from the start is critical to ensuring that DRM systems are not only acceptable but also address genuine gaps in care. We conducted qualitative interviews with a large, diverse sample of individuals with psychosis, recruited from multiple National Health Service (NHS) mental health services across 6 distinct UK regions. Interviews explored participants’ hypothetical views on a DRM system combining ASM and passive sensing, with data from both sources contributing to an algorithm to support relapse prediction. Given the breadth and richness of the interview data, findings for each DRM component (ASM, passive sensing [25], and relapse prediction algorithm) are reported separately across three publications.

This paper focuses on the views of people with psychosis about using an ML-generated relapse prediction algorithm as part of their clinical care, including potential benefits and risks of sharing algorithmic predictions with clinical teams, family members, and significant others. While previous studies have focused on clinicians’ views on ML in mental health care [26-29], only 1 study has qualitatively examined the views of people with psychosis on artificial intelligence (AI)–driven relapse prediction [30]. Unlike that study, which primarily gathered insights from online support group members regarding Facebook (Meta) data, this study provides the first in-depth examination of the views of people with psychosis about potential challenges and benefits of using a relapse prediction algorithm based on ASM and passive sensing data.

## Methods

### Design

This qualitative interview study formed part of the broader CONNECT project, a multisite research program funded by the Wellcome Trust [31]. Conducted during phase 1 of the program, it aimed to inform the design of the CONNECT DRM system. This manuscript adheres to the COREQ (Consolidated Criteria for Reporting Qualitative Research) guidelines (Checklist 1) [32].

### Ethical Considerations

Ethical approval was secured through the Health Research Authority and West of Scotland 4 Research Ethics Committee (reference 22/WS/0083). All participants gave informed consent, either in writing (signed form) or verbally (audio-recorded), and were assigned unique identification numbers to protect anonymity. They were compensated £20 (equivalent to US $24.74 at the time of the study) for their time.

### Participants, Sampling Strategy, and Recruitment

Recruitment occurred between November 15, 2022, and November 13, 2023, using the following inclusion criteria: (1) English fluency, (2) age ≥16 years, (3) current contact with mental health services, (4) capacity to provide informed consent, (5) clinical stability sufficient to participate in an interview (based on clinician or researcher judgment), and (6) either a formal diagnosis of schizophrenia spectrum disorder (*ICD-10* [*International Classification of Diseases, Tenth Revision*] F20–29 [33]) or fulfillment of early intervention service entry criteria, operationalized using the Positive and Negative Syndrome Scale [34] and/or the Comprehensive Assessment of At-Risk Mental States psychosis transition criteria [35].

A purposive sampling strategy was implemented to ensure diversity in geography, age, gender, ethnicity, type of service, clinical profiles, and previous exposure to digital health technologies. Participants were recruited from mental health services across 6 geographically distinct areas in the United Kingdom (Manchester, Glasgow, Edinburgh, London, Sussex, and Wales). Rather than aiming for data saturation, we aimed to recruit approximately 10 individuals per site to ensure broad geographic coverage and a large overall sample. As Braun and Clarke argue [36], data saturation is not relevant to all types of thematic analysis, particularly reflexive thematic analysis, used in this study. The specific target of 10 participants per site was a pragmatic decision; we considered this a feasible number to recruit with the time and resources available.

Clinical staff from acute inpatient units and community-based mental health teams (eg, Community Mental Health Teams, Early Intervention Teams, and Crisis Resolution and Home Treatment Teams) were asked to identify eligible individuals and provide them with initial information about the study (verbally or using a study leaflet). Interested individuals could then contact the researcher directly or provide verbal or written consent for clinical staff to pass their contact details to the research team. Study adverts were also displayed in secondary care mental health services and sent to participants from existing research cohorts, or other ethically approved studies, who had given prior consent to be contacted about research. The study advert invited interested individuals to contact the researcher directly. Research staff then provided all interested individuals with further information about the study, including a participant information sheet, verbal explanation, and the opportunity to clarify questions or concerns. Potential participants were given at least 24 hours to consider the information before deciding whether to participate. Those choosing to participate were then asked to provide formal consent via a paper, online, or emailed consent form or via an audio-recorded consent statement before the interview.

### Qualitative Interviews

Each consenting participant took part in a single, audio-recorded, semistructured interview, conducted in person, by telephone, or through secure videoconferencing, depending on individual preference and safety considerations. In addition to questions for eliciting participants’ views, the topic guide (S1 in Multimedia Appendix 1, and Figure S1a, Figure S1b, and Figure S1c in Multimedia Appendix 1) included plain-language explanations of key concepts, such as how an ML algorithm would be developed and used within a DRM system to predict relapse. Explanations included examples of well-known platforms that use ML algorithms (eg, YouTube [Google] and Netflix), a step-by-step lay description of supervised ML learning procedures, and a description of using the algorithm to alert clinicians of imminent relapse risk. Figures S1b and S2c in Multimedia Appendix 1 were shown to the participant at a relevant point in the interview; depending on the interview format, they were shown on paper (in person), via screensharing (video call), or posted or emailed ahead of the interview (phone call). Interview duration ranged from 20 to 78 (median 43) minutes. Participants also completed a demographic questionnaire. Interviewers completed short postinterview notes reflecting on context and recommending any necessary adaptations to the guide. All audio data were transcribed verbatim, anonymized, and securely stored.

### Data Analysis

Interview transcripts were analyzed inductively, using standard reflexive thematic analysis steps [37]: (1) data familiarization, (2) code generation, (3) initial theme creation, (4) revision of themes, (5) defining final themes, and (6) report writing. While coding was primarily data-driven, a subset of questions reflecting specific considerations (eg, whether participants would want family to receive algorithm-generated relapse alerts) was explored deductively, a common approach within digital health research [38]. Transcript excerpts were also systematically labeled according to whether views expressed related specifically to the ML algorithm, or to other aspects of the DRM system; this paper reports themes related to ML. Analysis was supported by NVivo software (Lumivero) [39]. The first author (EE) conducted primary coding, incorporating coding contributions from 4 Lived Experience Advisory Panel (LEAP) members and 2 McPin supervisors (AELW and AK), who examined a subset of transcripts. As several individuals coded the same interview transcripts, they met to compare their coding and resolve any discrepancies. Theme development was collaborative, shaped through discussions with (and written feedback from) the LEAP members and the broader research group to ensure credibility, practical relevance, and consistency with participants’ accounts. The study was grounded in a critical realist perspective [40,41], further details of which are available in S3 in Multimedia Appendix 1, alongside reflexivity details.

### Lived Experience Involvement

Lived experience involvement, supervised and coordinated by AELW and AK at The McPin Foundation [42], is central to the CONNECT study. A key component is the CONNECT LEAP, comprising 12 individuals with personal or familial experience of psychosis, drawn from the 6 study sites. This panel contributed to ongoing project governance and reviewed a summary of the study’s findings. All LEAP members were invited to attend a 2-hour introduction to qualitative methods training session, facilitated by McPin, outlining qualitative research methods and introducing the 6 key steps of thematic analysis. In total, 6 individuals with lived experience of psychosis contributed more substantively to the study design and analysis. Of these, 2 individuals read a draft of the topic guide and provided detailed written and verbal feedback to improve the clarity of lay explanations and interview prompts before use in the interviews; the other 4 were involved directly in qualitative analysis, with 3 being coauthors on this paper (one did not want to be named). After practicing coding in a synchronous session with the researchers and McPin supervisors, these individuals independently coded 3 interview transcripts. In 3 further synchronous sessions, they fed back their coding insights, advised on broader code development, and critiqued draft themes and emerging interpretations. Finally, they commented on drafts of this manuscript.

## Results

### Overview

**Figure 1.**
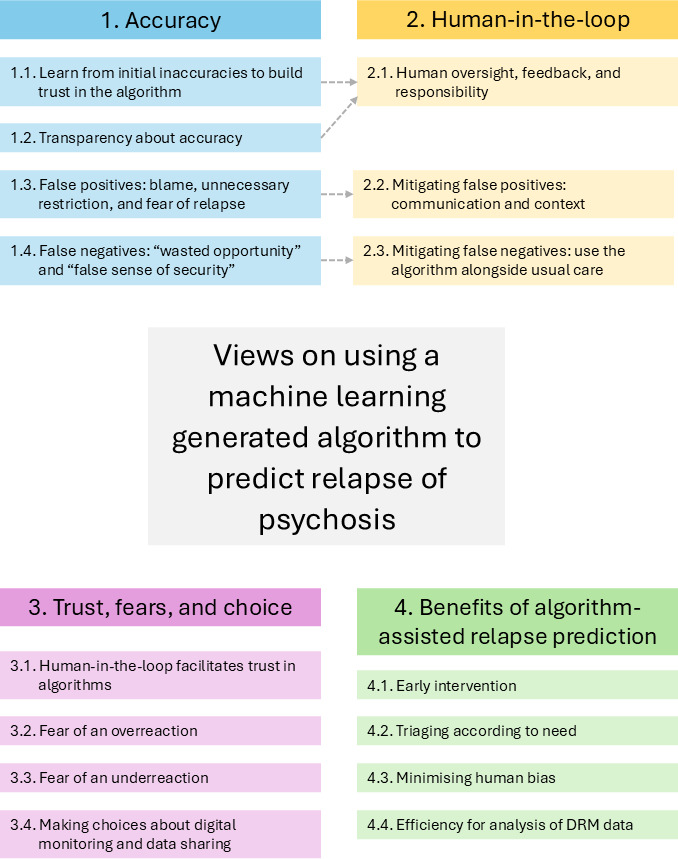
Thematic map.

Participants (n=58) had a mean age of 39.1 (SD 14.1) years. Just over half were men (n=32), a third (n=20) were from an ethnic minority group, and three-quarters (n=42) were single. Around half (n=27) were employed, in voluntary work, or studying, and around three-quarters (n=41) had received education after 16 years of age. Primary self-reported diagnosis was typically psychosis or schizophrenia, with current care usually community-based. S3 in Multimedia Appendix 1 illustrates full characteristics. Interview findings are described across 4 overarching themes, each with several subthemes, as summarized in Figure 1 and outlined below with selected quotations. S4 in Multimedia Appendix 1 provides the full coding tree, with additional quotations. For context, participant identification numbers that accompany the quotations are prefaced by a letter indicating their geographical region: Manchester (M), Glasgow (G), Edinburgh (E), London (K), Sussex (S), and Wales (C).

First, *accuracy* was a prominent theme, with participants discussing the risks of the relapse prediction algorithm producing false positives (flagging that someone was relapsing when they were not) and false negatives (missing actual relapses). In both cases, participants said that errors may be partially mitigated through a *human-in-the-loop* approach (theme 2), where DRM is blended with clinical oversight. The third theme, *trust, fears, and choice,* notes the interplay between users’ trust in the DRM system and their relationship with the clinical team. This theme describes participants’ fears about potential over- or underreactions from the clinical team in response to algorithm-generated relapse predictions. It emphasizes the importance of retaining choice around the use of relapse detection algorithms and the sharing of personal data. The final theme describes participants’ views about the *benefits of using a relapse prediction algorithm*.

### Theme 1: Accuracy

#### Learn From Initial Inaccuracies to Build Trust in the Algorithm

When discussing the development and use of relapse prediction algorithms, participants expressed some tolerance for errors early on. Initial inaccuracies were often considered “inevitable” (M011) and “understandable” (E007). Participants acknowledged that “it would take time…to perfect [the algorithm], so there would obviously be mistakes in the infancy of it” (E007), but generally accepted this as a normal part of software development and refinement:

*I suppose if any like, um, new Software or or new data management system, there are going to be teething problems.*

[C005]

*You don’t get everything right…But eventually it will it’s just taking a while, like it took YouTube years to get its algorithm right.*

[C007]

However, participants also conveyed a strong expectation that the algorithm should get more accurate over time, acknowledging that, to achieve this, “it’ll probably have to go through some sort of trial period” (K004):

*…it’s in its early stages of working, there is gonna be problems…unless you work on it and iron out the problems…And you can only do that once you start to collect the information.*

[E004]

Participants emphasized that repeated inaccuracies in the relapse prediction algorithm could undermine users’ trust in the system over time. One participant likened it to “…the boy who cried wolf!” (S002), suggesting that too many false alarms would cause both people with psychosis and clinicians to lose confidence in the algorithm’s reliability and disregard its alerts:

*It’d put doubt in their head…with people with mental health it’s all about that trust…so instantly that trust is lost.*

[C007]

*If [clinicians] then get sent this every single day and sometimes it’s misinformation as well, they’re just probably going to be reluctant to even take note of it…it might come through but they might be like, “oh, you know, this program, it’s not worked before, so how can we sort of trust it?” So I think…trusting the actual technology is really important.*

[C002]

One participant questioned the value of using an algorithm-based system at all if it fails to detect, or misrepresents, key information:

*If the program makes up or doesn’t catch information, then why are you working with the program if it doesn’t help?*

[K010]

Another reflected on their experience with a commercially available wearable device’s stress-detection feature, noting that repeated inaccuracies eventually led them to stop using it:

*I do just not really engage with it, so I do wonder if it would be similar [if the CONNECT algorithm was inaccurate]. If I might just feel, this isn’t useful, so maybe stop engaging.*

[G001]

Some participants stressed that the system should be “rigorously tested” (K003) and “trained trained trained trained trained” (S002) due to potential impacts on health:

*This is a very serious issue, so it should be checked checked checked*.

[S002]

*This is serious, it’s schizophrenia…it’s people’s mental health and it’s not a joke, is it?*

[S002]

*[Errors] would stop me from using it… ’cos that would be a kink in the system. It would be my whole health, mental health as well as my physical health and everything else…iPhones are a communication device. A breakdown in communication, that would stop me from using it.*

[E003]

Similarly, a few would only trust the algorithm if it was “fail proof: mistakes cannae happen” (G003), with a minority remarking that algorithm-based systems could *never* be accurate enough for them to trust:

*At some point this will lead to miscommunication or misunderstanding…The app is meant to report how your relapse is or how you’re feeling at the time. I would like to say there would never be an accurate communicative line through any form of apps.*

[M002]

#### Transparency About Accuracy

To foster realistic expectations and maintain trust, participants emphasized that people with psychosis and clinicians should be informed up-front of the algorithm’s inherent (known) limitations:

*Maybe even a statement saying it might not be 100% accurate but the app is continuously trying to make improvements.*

[C004]

*Realistically everyone should know it’s not always gonna get it right, and as long as we have that honest conversation and say…if this was to happen then we can, can talk about it.*

[M011]

Hence, if DRM users are aware of the algorithm’s fallibility from the outset, they may be less likely to lose trust in, or abandon, the system if errors occur. Participants underlined the need for transparent, pre-established protocols and procedures outlining how errors will be communicated and managed, ensuring users know what to expect:

*I think that should be shared with people using it, that it might not get things right and how to go about dealing with that when it doesn’t get things right.*

[K004]

Participants overwhelmingly recommended prompt, transparent communication if errors do occur: “tell them…straight away” (M010), be “open and honest” (C007), and “come clean” (K009) about the error. The exact format of communication was considered unimportant:

*...whether it’s a phone call, text or letter…as long as you can tell someone as soon as possible…the way you tell them is sort of up for debate really.*

[M010]

Participants underlined a need to “obviously apologize” (K009), “take responsibility…cos that person’s care has been affected,” and to consider the individual’s feelings about the error, “reassuring the actual person involved” (K009), “keeping channels open, and maybe actively leaning in, how people are finding things” (G001). Finally, the individual should be told what will happen in response; for example, “retraining” (K001) the algorithm, or taking steps to ensure that “it’s not something that’s gonna go on your record” (M011). The latter participant was very clear that they would only agree to use DRM if they knew that inaccurate information would not be kept long-term (eg, in their electronic health record). Hence, if the relapse prediction algorithm’s output is routinely transferred to health records, a robust and reliable procedure to correct inaccuracies would be crucial.

#### False Positives: Blame, Unnecessary Restriction, and Fear of Relapse

Participants’ varied expectations about how alerts from an algorithm-based system would be managed and interpreted appeared to underpin their opinions on how problematic false positives would be. If a human did not oversee the relapse prediction algorithm, or alerts were accepted uncritically by clinicians as “truth,” participants worried that false positives would result in DRM users feeling “blamed or singled out for something that I haven’t done” (E009), being told “there was a big risk or a big danger when there wasn’t” (K001), receiving unnecessary treatment or restriction (refer to “Fear of Overreaction” section), and experiencing fear of relapse. Conversely, those who expected that algorithm-generated alerts would lead to a thoughtful interaction with a trusted other were less concerned about possible false positives (refer to “Mitigating False Positives” section).

Fear of relapse was a concern for some, particularly in relation to the potential psychological impact of algorithm-generated predictions. Some worried that false positives could cause a “feedback loop” (S008) or “self-fulfilling prophecy” (K001), whereby being incorrectly told they were relapsing might generate unnecessary stress, prompt a fear of relapse, and potentially trigger actual deterioration:

*If it said you were going to relapse when you actually weren’t, you might actually get into your head like ‘oh I’m going to relapse’ and then you’re expecting it to happen…It’s more likely to happen when you’re thinking about it like that. When you’ve got it on your mind constantly.*

[M012]

Some participants preferred not to receive relapse alerts directly from the app to avoid potential distress caused by false positives (refer to “Making Choices About Digital Monitoring and Data Sharing” section). They preferred alerts to be routed first to a clinician or a trusted other, who could decide whether and how to communicate the information, reflecting the desire for a human-in-the-loop mediation to reduce distress and contextualize algorithmic outputs.

*That’s one of the reasons I think they should contact the, the mental health team and then them contact my trusted ones, because seeing my mental health is ok and then have app tells me your mental health’s not ok…my mind start racing thinking what am I doing wrong, why is this prompting me…So saying my mental health wasn’t ok, I would like to know through a person.*

[G005]

Conversely, despite initial worry, 1 participant described feeling confident that, with time, they could judge themselves whether they were unwell:

*I would be really concerned…But I think I know myself enough that praps after a day or so and I’m still alright…I’d know.*

[S007]

Others would find false positives unconcerning and would appreciate the “warning” (G008) as a prompt to “check in” (M005), “think twice” (M010), and pay more attention to their mental health:

*…probably I’m just, will be more alert to my situation…sometimes it’s good to have the alert even though it’s might be not accurate.*

[G009]

#### False Negatives: “Wasted Opportunity” and “False Sense of Security”

The value of a digital relapse prediction system lies in its ability to detect early warning signs *before* an individual necessarily becomes aware of them. If an individual notices their own relapse signs first, participants felt that the system is “not really doing the job it was designed for” (G007):

*I want to know if there is going to be a relapse, because then I can do something. If I don’t know, it’s a wasted opportunity… If I know beforehand, I could make a real difference to outcomes and experiences for myself.*

[K001]

Hence, false negatives were a concern because of the missed opportunity to intervene early in the relapse process, meaning that “people are falling through the gaps” (S007). Participants noted that intervening early could improve outcomes, but delays from false negatives were potentially harmful, allowing symptoms to escalate unchecked and increasing the risk of a more severe episode:

*[False negatives are] worse for the…person in the long run. They could become very ill, you know, nothing’s done but it could be too late before any action’s taken.*

[K009]

Similarly, participants warned that an algorithm that generated false negatives may provide “a false sense of security” (E009) whereby “you would keep getting worse, thinking you were getting better” (G005). This may delay help-seeking until a point at which individuals are no longer willing to seek help:

*...you might get too deep into it and not want to reach out anymore.*

[M012]

One participant, whose voices did not like her talking about her mental health, noted that false negatives would be counterproductive because they would “reinforce…what the voices are saying…what they want” (S007). Moreover, false negatives would waste the user’s efforts to track their symptoms:

*If you’ve filled all these specific questions and gone through a specific process…the whole point of it is…to pick up on [signs of relapse] before you do, so if you realise a bit before the computer has, it’s almost pointless and…that would eliminate the purpose of it in the first place.*

[M010]

### Theme 2: Human-in-the-Loop

#### Overview

Participants frequently advocated a human-in-the-loop workflow to improve the algorithm’s accuracy (refer to “Human Oversight, Feedback, and Responsibility” section) and to manage the potential consequences of false positives (refer to “Mitigating False Positives” section) and false negatives (refer to “Mitigating False Negatives” section).

#### Human Oversight, Feedback, and Responsibility

Participants emphasized the importance of “human guidance and supervision” (C005), “safeguards” (C005), or “human oversight” (C005) for the relapse prediction algorithm. They suggested incorporating a feedback mechanism to allow the individual with psychosis, clinicians, or trusted others to provide information on the algorithm’s accuracy and to “calibrate” (G005) it where necessary:

*A facility for maybe friends or family…to question whether it’s right or not. Or…the individual, if they’re in the right frame of mind…could give feedback…If you do feel that [the alerts are] out of sync with what is actually going on with you as an individual, give us feedback and let us know.*

[C005]

Being able to provide feedback themselves would help some participants feel comfortable using an algorithm-based system. For example, participant G001 was willing to try a DRM system “as long as I felt I was able to have my view taken into account.” Others saw a feedback mechanism as just “a very practical thing” (G002) to help train the algorithm and improve accuracy.

Participants generally assumed that their clinician would oversee algorithm-generated alerts and provide feedback on their accuracy, integrating this process into usual care. Although this arrangement would have the advantage that alerts could be overseen by “people that know me” (C003), clinicians may not know how to interpret the data:

*The odds of your mental health team knowing everything about CONNECT and how to interpret data are probably quite small. I would rather somebody that actually knows about the data and what that can or can’t say.*

[G001]

Hence, several participants suggested that algorithm-generated alerts should be automatically sent to a dedicated clinical review team, whose deliberations would confirm or refute each alert (or a subset):

*You should employ people to monitor that…So whenever the program makes a discovery or a pattern, that gets notificated to the team that you make, and then that team debates whether that is right or wrong…And then that helps the program work out things better as it goes along.*

[C006]

*If you’ve got self-learning AI or if you’re doing machine learning, then it’s more likely that those mistakes will be used to correct it. I think as long as you have a team that are evaluating it every x amount of days, weeks, then I think it’s fine.*

[M008]

*If you’ve got self-learning AI or if you’re doing machine learning, then it’s more likely that those mistakes will be used to correct it. I think as long as you have a team that are evaluating it every x amount of days, weeks, then I think it’s fine.*

[M008]

This “oversight committee” (C005) could safeguard decision-making, provide “an ethical and moral point of view” (C005), and supply iterative feedback that progressively improves the model’s accuracy. Participant C005 noted the potential ethical and political complexity around who should convene and manage such a committee. They suggested that, if a private company owned the DRM system, additional oversight by an external party would be needed:

*I would be slightly more concerned if it was…information being collected and then analyzed and decisions being made by private individuals, because…most CEOs and companies that own AI facilities are not [publicly] elected…so…if you are going to use AI machine learning there would need to be, um greater oversight via either like NGOs or you know have intergovernmental organizations just keeping an eye on it. If it is going to be run by private corporations.*

[C005]

This quote raises questions around who is ultimately responsible for such tools and their impacts. As one participant noted,

*There needs to be some human involvement…because…the computer program might make mistakes that a human would have to, someone would have to answer for.*

[K007]

#### Mitigating False Positives

##### “We Can Talk About It”

Participants who expected that algorithm-generated relapse alerts would be “supervised by a human” (E008) and used to prompt clinicians to gather further information tended to regard false positives as relatively unproblematic:

*If it thinks that I’m getting unwell and my mental health team get in contact…I can tell them that I’m fine.*

[M004]

Most participants thought that algorithm-generated alerts should prompt clinicians to contact them directly: “a wee telephone call fae your [community psychiatric nurse] asking you how you’re doing” (E004). They emphasized that this would facilitate “openness [which] is quite important to have” (G008) and that this contact could mitigate the effects of false positives by allowing clinicians to “talk to you…to kinda confirm what the data from the phone says” (E007). Participants were clear that relapse algorithms should “not solely just being left to make decisions on its own” (C005). Instead, there was a strong sense that participants viewed algorithm-generated alerts as provisional information, to be confirmed by direct “human interaction” (S002):

*If the…technology suspects there is something wrong then you should be seeing the psychiatrist, or the psychiatric nurse to make a further assessment to check.*

[E008]

*The app can be a way to notify someone that there’s an issue and then the person will come in and you can have that discussion.*

[M005]

Consequently, several participants considered false positives to be less concerning than false negatives because “obviously if it picks it up and it’s wrong…we can talk about that” (S001). However, one of the same participants noted that the person with psychosis may not always be aware that they are relapsing, creating ambiguity as to whether the algorithm or the person themselves is correct. This reflects the need for collaborative interpretation between the individual and their clinician to negotiate meaning and action when predictions arise:

*I’d have to sit down with the doctor, explain to them why I’m not feeling like that and hope they understand, really. Or I need to face facts that I am getting ill and the computer is right, maybe it’s not wrong.*

[S001]

Moreover, while some participants acknowledged that human oversight could help mitigate errors, others expressed concern that frequent false positives might undermine the system’s credibility, leading users to take the system less seriously over time (refer to “Learn From Initial Inaccuracies to Build Trust in the Algorithm” section). They also emphasized that long-term trust in the system would depend not only on the algorithm’s accuracy, but critically on the strength of the relationship between the person with psychosis and their clinical team (refer to “Human-In-The-Loop Facilitates Trust in Algorithms” section).

##### Contextualizing Passive Data

Participants frequently warned that relapse prediction algorithms that rely on decontextualized passively sensed data may be prone to false positives. They gave examples such as that of an elevated heart rate (readings from exercise could be misclassified as stress) or that prolonged time spent at home might be flagged as social withdrawal, even when unrelated to mental health. Participants suggested that the risk of false positives from passive sensing could be reduced if algorithmic outputs were reviewed by someone who knows the individual personally (ie, who has additional context):

*It’s tied to people that know me, so they’ll know better if I am not ok… for example, two or three times a week I’ll just sleep till like 1pm and I’m not relapsing or anything, I’m just enjoying a little break.*

[C003]

Interviews revealed a shared perception that relapse prediction algorithms based on passively sensed data were more prone to false positives than those using ASM data. Participants may view ASM as more reliable because it reflects self-reported experiences, offering direct insight into mental health status, whereas passive data can be ambiguous without context. Several participants suggested that existing (E004) or additional (G005) patient-reported ASM data could provide the context for passive data, helping to clarify when behavioral patterns were benign or clinically significant:

*…here’s my monthly input here for January, for example, and as you can see I’ve been fine so why is that highlighting that I’m needing help?*

[E004]

*About the phone knowing that I’m home…it could…pop up a message: “are you alright?…Are you enjoying staying at home or…staying home for another reason…that you’d like to share?*”

[G005]

In sum, accuracy could be improved by contextualizing passive data using subjective self-report, supplemented by confirmation or refutation by known others.

### Mitigating False Negatives

Some participants felt that false negatives were less concerning if an algorithm-based system was used alongside usual care, as “just an additional thing” (G009), rather than being relied on to replace clinical judgment:

*You can always seek help…if you know yourself. If you don’t know, then it’s a psychiatrist’s job to work it out, it’s not the machine’s job. It couldn’t be used as a replacement for a psychiatrist.*

[E001]

*If it misses you’re deteriorating then that would be the same if there wasn’t an app…it’s only offering things that are better than they’re currently there… it’s not making anything worse.*

[G008]

These participants considered it a bonus if an algorithm provided information that helped them spot relapses but did not want to entirely delegate responsibility to the system.

*As long as there’s not…a hundred per cent dependency and then there’s other avenues of help still available, I think it’s fine.*

[M008]

*It’s down to me really isn’t it to reach out, I can't rely on everything to do that for me.*

[S006]

### Theme 3: Trust, Fears, and Choice

#### Human-in-the-Loop Facilitates Trust in Algorithms

The nature of the relationship between the individual and their clinician appeared to play a key role in facilitating trust in algorithm-based systems. As participant E003 summarized,

*I think it can be trusted just by…trusting the persons that’s using it. That’s the only way you can trust it. You have to be able to trust the person.*

[E003]

Similarly, the first author’s analysis diary (detailed notes written during coding and analysis) noted:

*The relationship with the care team is key. It takes a long time to build trust.*

[analysis diary: G003]

*It’s like the app is an extension of the relationship with the team, so the willingness to engage is directly affected by the relationship with the team.*

[analysis diary: general]

Three relational factors emerged as pivotal: (1) mutual trust, (2) the clinician’s stance (collaborative vs authoritative), and (3) the individual’s previous experiences of coercive or restrictive care. Mutual trust was repeatedly cited as crucial, particularly for handling false-positive alerts:

*I know myself more than anyone else, so they would know my word.*

[C003]

*I think if your team checked in with you…I trust them…I trust them.*

[G007]

Participants who had already established a trusting and collaborative relationship with their clinical team were more likely to perceive algorithm-generated alerts as helpful and were less concerned about occasional errors. Conversely, participants who had experienced strained therapeutic relationships, been misunderstood by the clinical team, or been exposed to restrictive practices in the past appeared less willing to place trust in the use of algorithms in their clinical care. For example, participant E009 spoke of “mistrust,” “suspicion of the psychiatrist and the mental health team,” and a desire to “keep them off my back,” linking these feelings to previous experiences of coercive care.

*I’ve experienced the…bad end of it with sections and depots and…enforced things…When it got nasty with the sections and things that’s when I started to think differently.*

[E009]

This participant repeatedly warned that an algorithm might “incriminate me” or provide “ammunition” for clinicians:

*I wouldn’t like to incriminate myself. I wouldn’t like [to] think we were on the same page and then one day they turn around to me and say “oh we’re concerned about you because of this that or the next thing that we’ve…machine learned.*”

[E009]

This participant conveyed clear concern that an opaque relapse prediction alert could potentially disrupt their carefully cultivated therapeutic alliance. Noting that their alliance had recently improved, they began to tentatively express that DRM might be beneficial in this context, but immediately reconsidered, reiterating their worries about trust:

*It might [be beneficial], I mean if you’re on the same page with them like I am now, but you know like I said it’s trust issue, trust and suspicion and mistrust you know.*

[E009]

Similarly, another participant (C001) used less emotive terms but contrasted 2 mental health teams they had received care from one they perceived to be helpful and supportive, and the other “didn’t give me the support, but they felt… what was going on with me either it was my medication or hospital,” (C001). This participant anticipated that using DRM with the first team would be useful, but with the latter, it would have caused more restrictive care (hospital). These accounts highlight the influential role of the patient-clinician relationship in shaping perceptions of algorithm-driven monitoring. They provide context for the following subthemes, in which participants anticipated 2 contrasting responses to relapse prediction alerts—an overreaction, characterized by increased restrictions and control imposed by clinicians (refer to “Fear of an Overreaction” section), and an underreaction, where alerts are overlooked and no meaningful support is provided (refer to “Fear of an Underreaction” section).

#### Fear of an Overreaction

Some participants feared that algorithm-generated alerts would prompt “an overreaction and, in the context of mental health, an overreaction can mean unnecessary restriction and you really want to avoid that” (K001). Specific anticipated responses included enforced rehospitalization and/or medication increase:

*I just don’t want to be sectioned again…it’s really hard to get out when you’re sectioned.*

[G007]

*If they felt they needed to then they could…double my medication you know.*

[E009]

When discussing these fears, participants often alluded to the power they perceived clinicians to have over their lives, using phrases that expressed their own felt lack of power (“against my will” [C001]) and their strong feelings on the topic (“would put me in a panic” [G002]). They also described previous experiences of hospitalization (“really distressing” [E004]) or medication side effects (“feeling tired and everything like that” [E009]), underlining the impact of feared overreactions.

Given the potential for clinicians to assert this power in response to algorithm-generated alerts, it is unsurprising that trust was also a key factor here. Individuals who trust that clinicians will carefully weigh algorithm-generated information alongside other evidence, including evidence that they seek directly from the individual themselves, may be less likely to fear an overreaction:

*They need to speak to the individual and find out what they are struggling with. It needs to be communicated in a way that they both feel safe, and it doesn’t feel like the individual or the service is going behind each other’s backs…They can’t just take charge from it.*

[C004]

In this and other quotes, participants contrasted 2 hypothetical experiences of algorithms; first, receiving direct, collaborative communication from the clinical team, and second, receiving an unwanted escalation of care in which the individual feels surprised and out of control.

Fears of clinicians’ overreactions were particularly prominent when participants discussed false positives. However, they were also sometimes mentioned when describing true positives, with challenges arising specifically when there was a discrepancy between what the DRM system reported and what the individual wanted to convey to clinicians themselves (“Only if it’s contrary to what I was saying” [G002]). For example, they may wish to conceal a deterioration from clinicians:

*Sometimes it’s best not to give all the information to psychiatrists.*

[E009]

*As long as someone’s honest if [the alert is] wrong or if it’s right, I think it’s fine, but then that brings, like, the other complication…if they are being honest or not.*

[M010]

Alternatively, an individual may not realize or believe that they are unwell. Hence, even if DRM alerts prompt clinicians to speak to the individual directly, this might be an unwanted surprise; unraveling whether an escalation in care is required may not be straightforward. However, the challenge of weighing competing evidence about relapse is not unique to algorithm-generated data, as this example highlights:

*Would you be informed it was going to be going to your care team?…Because the one day that I was really stressed was when my mum phoned my care team, did nae phone me and the next minute I had three of them waiting to section me.*

[E004]

*My mum…got the wrong end of the stick but she had to write a letter to the hospital to explain she’d got the wrong end of the stick before I was getting out…It was awful.*

[E004]

#### Fear of an Underreaction

Some participants expressed fears that clinicians would not act on information provided by algorithm-generated relapse alerts due to viewing alerts as unimportant (“if was up to them, the mental health team would do nothing” [G002]) or inauthentic (“I’m unsure about how people would perceive it’s authenticness…If people would kind of take that information on the results seriously” [K003]), especially if they were not always accurate. Others worried that receiving data from the system would increase clinicians’ already large workload (“Does it not make the Doctor a lot busier?” [E003]) and that many would simply be too busy to act on the information.

*I think it would be really, really practical. But I know it won’t happen, it’s not going to happen at all. It’s like you cannae get a reading to the doctors, you’ve got to phone in and see what they’ve given you on the phone.*

[G010]

*Already they’re stretched with what they’re doing and then they’ve got to attend to these messages as well…So, I don’t think they’d be acted on very quickly…by the clinical team…And so, you’ll be left with it for days, thinking about your appointment.*

[K002]

The lack of human resources to follow up on digital alerts was a particularly pressing issue for participants. K002, who repeatedly emphasized that a digital monitoring system would not scale well to real-life services, that the clinical team would not respond promptly to digital health data or predictive system outputs, and that this lack of response may stop people using the system and negatively impact individuals with psychosis. The latter concern was echoed by M011:

*I think you’ve really got to work on the response and whether it’s going to be suitable for a large-scale rollout…If you don’t have that in place, people are going to get frustrated, and they won’t use it.*

[K002]

*Maybe me feeling like somebody should help me out…I should receive a little bit of additional support and not getting that and then feeling that rejection*.

[M011]

Several participants emphasized the need to carefully consider how the system would be implemented to avoid being “…overwhelming for all the workers” (C004). Suggestions included restricting the use of digital monitoring to certain days, distributing review of DRM data across staff members, limiting system checks to working hours, allocating dedicated time for staff to review digitally generated data, or establishing a dedicated digital review team (refer to “Human Oversight, Feedback, and Responsibility” section).

#### Making Choices About Digital Monitoring and Data Sharing

##### Choice About Using a Relapse Prediction Algorithm

There was near-unanimous agreement that using a DRM system incorporating a relapse prediction algorithm should be “an individual choice” (G010), and a clear consensus that making access to mental health care conditional on DRM use would be inappropriate:

*I disagree with…gating access to mental health. I think there should be as few limitations as possible to access mental healthcare.*

[M008]

*I don’t think it should be mandatory, I think it should be voluntary, and I don’t think it should be tied into whether or not you get care. I would suggest that’s unethical.*

[K001]

*I’d be devastated if [it was compulsory]. It’s a terrible idea. It’s taking away the person’s independence. It’s treating them like a robot controlled by a computer. It’s…being blackmailed, that if you don’t use this device, then you don’t get treatment. It’s a terrible idea.*

[M003]

Participants expressed strong feelings on the topic, citing practical (“not everyone has a smartphone and a wearable” [M004]), clinical (“people might be paranoid about the machine” [E008]), and moral reasons (“sounds like a human rights violation” [G001]) against making DRM mandatory.

##### Choice About Sharing Algorithm-Generated Relapse Alerts With the Clinical Team

As well as having a choice to use a DRM system, participants noted that users must have a genuine choice about whether to share algorithm-generated relapse alerts, and with whom. There was some apprehension that an individual’s decision not to share alerts with clinicians may be pathologized and could result in them being labeled as noncompliant.

*The program I suppose is harmless. I just, I don’t really want the information relayed back to a professional…it’s not all dependent on a mental illness. Some of it’s just choice I think.*

[E004]

*I just don’t feel anything should be mandatory in mental health because usually there will be somebody it doesn’t work for, then they will just be labelled with treatment resistance or…not willing to engage…You see that with anti-psychotics already and it would just be crap if this becomes like a new… It is good to have options.*

[G001]

Participant G001’s reflection highlights a wider concern that DRM may replicate existing dynamics of coercion in mental health care. Drawing parallels with antipsychotic medication, they cautioned against digital tools becoming mandatory or default components of treatment, emphasizing that a one-size-fits-all approach risks marginalizing individuals for whom such tools are ineffective or unwelcome. As with antipsychotic medication, there is a danger that nonengagement with DRM could be mischaracterized as resistance or noncompliance, rather than understood in the context of individual needs and preferences. These concerns underline participants’ broader views that digital tools should be offered as optional and flexible components of care, embedded within a framework that prioritizes autonomy, choice, and collaborative decision-making.

Participants displayed a range of views about whether they, personally, would want to share alerts from the relapse prediction algorithm with clinicians. Some were clear that they would not want to share their DRM data (“I wouldn’t do it. Yeah. I don’t feel desperate enough.” [K002]), whereas others were optimistic about its potential benefits (“If it can tell them what to do, that would be fantastic.” [K010]). Several indicated they would be willing to share alerts if they were also notified (“If my mental health was tipping and it was gonna notify someone, it would be good to know first.” [C006]). Some noted they were already “quite open with my mental health team” (M004), so sharing DRM data would not be a concern. Others were more ambivalent; for example, despite being receptive to DRM and acknowledging its potential benefits, participant K004 preferred to show clinicians the data in person:

*You could share that with them at a meeting or appointment…because then it’s like a two-way conversation…Yeah, I guess that takes away the automatic side of it, and I don’t know, that could be a downfall as well. Because it’s in some peoples’ cases, if things are getting really bad, then it is best for someone else to get involved, but I think you have to look at it case by case.*

[K004]

Overall, more participants expressed willingness than resistance, despite worrying about an overreaction (refer to “Fear of an Overreaction” section) or underreaction (refer to “Fear of an Underreaction” section). Again, the level of trust with clinicians was a key factor. Participant G001 preferred not to send DRM data to clinicians, noting that “I would need to be very bad indeed before I went for help from the team…I would prefer more tolerance to stuff before information was passed on” (G001). Accordingly, this individual preferred DRM data to go “through some kind of filter first, like maybe somebody from the [research] team could maybe check in to see if there is actually a problem” (G001).

##### Sharing Algorithm-Generated Relapse Alerts With Family Members or Carers

When asked whether they would share relapse alerts with family, carers, or trusted others, participants repeatedly emphasized that consent from the person with psychosis is essential. Many agreed that “if it was with permission then…it’s completely fine” (M008). As with clinicians, the nature of the relationship between the person with psychosis and their family, carer, or significant other was key to their willingness to share data. Participants would share algorithm-generated alerts with “people that I trust” (G005) but cautioned that it was necessary to be “aware of the family dynamics” (C005). Not everyone would have a good enough relationship with their family to share data:

*Some family don’t talk to each other and they don’t care.*

[K006]

Doing so without a supportive relationship could be counterproductive:

*…if they are in close contact and they’re on good terms. I think that’s the key term there would be good terms, um because imagine if you didn’t have a very supportive family, yeah I suppose you will feel like they’re constantly interfering. Intervening when you wish they weren’t.*

[C005]

Some participants would need to think carefully before sharing relapse alerts with certain supportive others because alerts “could upset them” (C006), cause stress, or might affect the relationship:

*It might be quite frightening for her to get a thing saying her husband’s mood’s low…and he’s thinking of…suicide, I think that’d be quite a big thing to send through a text or an app…Cos she was upset even when we talk about stuff that I did in the past…so if she started getting pings about how low my mood is, I don’t know.*

[G007]

*If they had information about me…which the doctors and nurses had…it would just be like…cheating on me…paranoid in the relationship - don’t want that.*

[E003]

One participant noted that their family was not geographically nearby, so receiving alerts might “put them in such a panic, because they are not here, they are not seeing me” (M006), whereas “if it’s a relative that is close by, like, easily just driving, and just see you face-to-face, and see for themselves, yeah, that’s fine” (M006). This was deemed particularly important if there might be errors in the algorithm:

*When somebody sees you face to face they can say, oh that must really be an error*.

[M006]

Similarly, participant K001 emphasized that all recipients would need information on the algorithm’s accuracy so that “they knew how reliable it was.”

Nevertheless, if these concerns were sufficiently addressed, participants identified several potential benefits of sharing algorithm-generated alerts with a suitable person. Relapse alerts “maybe could spark a conversation” (G001), provide “a good bonding point for us” (G010), “would make life easier” (K010), and help the recipient better understand the DRM user’s experiences:

*I think it would be a good idea…cos then she would kind of know what’s going on.*

[S005]

*It’ll be more helpful for the family members so they can look it up what my illness is like and what to do…what the triggers are on my mental health and stuff like that.*

[M007]

Participants hypothesized that receiving relapse alerts may be “be reassuring and comforting” (S006) to the family, carer, or trusted other, and to the person with psychosis themselves:

*I think that’s a really good idea again because carers are the ones that probably feel the most pressure of keeping somebody that’s suffering with complex mental health issues safe. I think they’re the ones that have that emotional investment as well, ’cus I have a carer and I don’t always share everything with her, but if the app alerted her…then it gives them a chance to maybe speak to somebody…sort of help me access the support that I need in that moment*.

[M011]

*I’d feel as if I’m being watched and looked over which is really good.*

[M014]

Finally, participants emphasized the value of involving trusted family members or carers (with consent) to help contextualize data generated through DRM and avoid fluctuations in behavior or mood being misinterpreted as clinically significant.

*If anything, it will be more appropriate for maybe close family and friends to get a message, if they consented…because…sometimes…things can be flagged that aren’t really that serious but are interpreted seriously, and then you’ve got a situation where you feel like you’re being watched or everything you do is going to cause people to flag a concern.*

[K004]

Nevertheless, others preferred clinicians to receive the alerts and then to contact family or carer, outside of the DRM system, to gain additional context:

*They could reach to people that are close to me and talk to them and try to understand if there is something going on.*

[G005]

*...and then of course contacting the person, like mys…me, but try to get the context before and see*.

[G005]

*Yeah, I don’t think that needs to go through to like an app or anything, I think it can be…as long as you made contact kinda thing that’s the main thing.*

[G008]

##### Receiving Algorithm-Generated Relapse Alerts Oneself

When considering whether they would wish to receive relapse alerts themselves, a few participants expressed concerns that alerts may generate “unnecessary stress” (M012), causing DRM users to “maybe get anxious” (E007), “get panicky” (E007), and “end up worse than they was before” (M012). Participants anticipated that even *correct* alerts (true positives) would sometimes prompt such fears (as well as false positives; refer to “False Positives: Blame, Unnecessary Restriction, and Fear of Relapse” section). Participant C005 warned that alerts “would have to be tailored to the conditions that people have” because “what works for some people might not work for others.” They hypothesized that people with certain diagnoses might find alerts especially challenging:

*If someone has schizophrenia or they’ve got severe tendency to repetitive psychotic episodes… I wouldn’t advise you getting an alert like we all did last weekend [UK emergency alert system test] on your phone because that would probably set some people off.*

[C005]

*If you were constantly notifying someone with anxiety that [their] behaviour seems a bit erratic, it might set them off*.

[C005]

To prevent or mitigate distress from true or false positive alerts sent to oneself, participants suggested carefully phrasing messages (M012) and/or filtering them first through clinicians, family, or trusted others (S001):

*Instead of saying you’re relapsing it could say “signs of relapse”…and then tell you some techniques and stuff you could use to like you know, pull yourself out of it.*

[M012]

*If anything goes wrong, let the doctor see it, they’re professionals at the end of the day. If they think that you need speaking to, then speak to you, but don’t…I wouldn’t send it to the patient every time, because…it could just be a minor problem…that’s set them off a bit.*

[S001]

Even with such adjustments, some individuals felt that direct alerts were unnecessary (“I know if I’m having a rough time” [K002]) or overly intrusive (“If you go through psychosis, the amount of input you get is a lot. And then to feel like it’s still being sort of jammed down your throat would just be quite, yeah, intense I think,” C002). Similarly, although open to receiving alerts, one participant worried that alerts may prompt him to switch off his phone in some circumstances:

*As an idea it’s amazing…but if I’m thinking “well no sod everyone and everything” then it might make me switch my phone off even more, and had I not been notified I wouldn’t have done…I think it depends on each situation and each individual and their tolerance levels.*

[M011]

Conversely, many participants were enthusiastic about receiving algorithm-generated alerts, describing them as “a wake-up call” (C006), a “nudge” (E008, G008), a “chance to introspect a bit and… give you the kick to…pay a bit more attention to your own health” (M008). There was a shared view that alerts could compensate for difficulties in recognizing mental health deteriorations:

*Sometimes you’re the last one to be aware of your mental health deteriorating.*

[G008]

*It is maybe picking up on things you have maybe not noticed are tricky as well. It might make you see things in a new light potentially*.

[G001]

*You might not realise that something went wrong…So it would be good to have a like “oh hold on you’ve gotta stop doing that.”*

[C007]

Participants noted that alerts may be particularly beneficial for individuals with limited existing support (“For me…getting in there early was recognized by my family and friends, rather than a smartwatch. But for people who maybe are a bit more isolated…that would be helpful for them.” [C002]), for those living alone (“It’d be a bit of a relief because when you live on your own you don’t notice things.” [S007]) and for those facing abrupt relapses (“It’s just that’s going down and down and then the next minute you know it you’re on your knees.” [G006]).

Several interviewees spontaneously proposed additional messages that the DRM system could deliver to encourage help-seeking or support mental health.

*It would have like…“your mental health is a bit low, so these are things you could do to increase it, or people you can contact or steps you would take.”*

[G005]

*I think it needs to have sort of “what can the app do to help now, now that we recognize the signs?” Can you do something like self-care: do you want to try and brush your teeth; do you want to try and eat something today? Something that leads on from the app saying that we recognize your mental health is struggling, because it’s good that it states that, but it needs to make someone confident in the sense that they can do something*.

[C004]

Preferences for alert delivery format differed among individuals, highlighting the importance of tailoring format to personal context and communication habits:

*I think that varies from person to person…at the start maybe sign up for how [you] want notifications to come through…cos some people check their emails…some people never check their phones or texts…I probably want it…as a notification.*

[G008]

### Theme 4: Benefits of Algorithm-Assisted Relapse Prediction

Overall, participants highlighted 4 potential benefits of algorithm-assisted relapse prediction. First, they emphasized its value for early intervention, describing it as a “proactive” (E002) method that could identify relapses early and prompt individuals to “look after [themselves] better, or do things which [they] know help in the context of a relapse, or seek help” (K001). They anticipated that, by intervening early rather than having to “wait for things to get worse” (M006), they could avoid the disruption of a full relapse (“A way to stop me from going under” [M001]).

Second, participants noted that DRM could be used “like a triage” (E008), allowing “information to be passed on in a timely manner” so that clinicians could allocate resources according to need. They proposed that DRM evidence could speed up the process of getting an urgent doctor’s appointment (“You can’t always get in contact with your GP [General Practitioner] …usually you’re like seventeenth in the queue” [M011]), or inpatient bed (C007 quote), and be used to allocate other finite resources such as staff time (E004):

*If they could work alongside the wards, let’s say I had my psychotic episode, they might be able to say ‘look this is a priority we need to get this person in now’…They’d be able to prioritize who needs it more and who needs it less*.

[C007]

*If they knew everybody was on that [app] and they’d just be getting…wee alerts “you better phone [name] today,” or “you better phone Pam down the road today”…so it would help them keep on top of their workload better.*

[E004]

Third, in contrast to others’ views that algorithmic relapse predictions might not be accurate (theme 1), some thought that ML might “mitigate that human error that you could get if it’s done by a person” (G008) or “eliminate human bias” (M008). Participants alluded to errors and biases originating both from service users themselves and from clinicians, noting the unequal power associated with these roles. For example, participant E002 described the DRM relapse prediction algorithm as “fantastic” as it may help redistribute power by facilitating direct communication:

*There’s miscommunication between the staff so sometimes the doctor will say something and it’s not true and I’m like “that’s not true” but they’re like “Well I’m the doctor and I’ve been told this” and I’m like “well you’ve been misinformed”…There’s an awful lot of people saying one thing and the doctor can say anything, they’re all powerful, the power goes to their head. I think this way is much more kinda wholesome even though its advanced technology, because its actually coming from you and there’s not a middle man who’s, for whatever motive they’ve got, sticking an oar in it… Eliminating human error and miscommunication is clearly the way forward here.*

[E002]

Fourth, ML was seen as an efficient way of analyzing extensive digital monitoring data: without ML, it “would take a long time to process this much of data” (G009), whereas “through machine learning it would be much quicker” (G009). Participants highlighted DRM’s value in “cutting down on worked hours for human people to actually have to go and be trained and then assess people and then tabulate all that” (M008). Particularly in the context of NHS staff shortages, ML was seen as valuable as it would “save the staff from having to do a job like [analyzing DRM data] and frees them up for other jobs” (M010), with one participant reflecting that health services have a “responsibility to use the most up-to-date methods to assess and treat people. So I think health services should use it as soon as it was verified and everything” (M008).

## Discussion

### Overview of Findings

This study explored the views of people with psychosis on using an algorithm-facilitated system to predict psychosis relapse and their perspectives on sharing relapse alerts with their clinical team, family members, or significant others. With its large, diverse sample, detailed topic guide, and strong lived experience input, it provides a comprehensive and nuanced understanding of stakeholder perspectives on the ethical, practical, and relational implications of using predictive technologies in mental health care. Accordingly, Tables 1-3 outline specific, detailed recommendations for implementation, based on the study’s findings.

**Table 1.** Accuracy: recommendations based on the study findings.

Finding	Recommendation
DRM^a^ users are likely to tolerate some level of errors in an algorithm-based system during a training phase but need to know how accurate to expect relapse predictions to be.	Inform all DRM users (people with psychosis, clinicians, family, or significant others) up-front of known limitations with the algorithm and what to do if errors occur.
Participants thought it important that algorithms get more accurate over time.	Build in a feedback mechanism so the algorithm learns from errors. For example, allow the clinical team, the individual with psychosis, or a dedicated digital review team to calibrate the system’s predictions.
Some participants considered false negatives worse than false positives because false positives could be discussed with the clinical team.	When calibrating the algorithm, developers may wish to favor increased sensitivity over specificity. However, if this is done, the balance of sensitivity and specificity should be clearly communicated to all DRM users.
DRM users may be less confident in algorithm-based relapse predictions that are based on passively sensed data than those based on ASM^b^ data. Users may perceive passive data as ambiguous without context.	Ensure that all DRM users have a clear, lay explanation of how the relapse prediction algorithm combines ASM and passive data to make predictions. Provide opportunities to seek additional information to allay concerns (eg, could a heart rate increase during exercise be misunderstood as relapse?).
DRM users value openness and honesty about errors in an algorithm-based system.	Be transparent about errors that occur and check their impact on DRM users. Provide reassurance and additional support to mitigate any additional anxiety caused.
Only accurate DRM data should be retained (eg, in health records).	If DRM data is automatically transferred to electronic health records, have a clear protocol for how to check data and correct inaccuracies; be clear who is responsible for ensuring accuracy of records.

a DRM: digital remote monitoring.

b ASM: active symptom monitoring.

**Table 2.** Human-in-the-loop: recommendations based on the study findings.

Finding	Recommendation
False positives are less likely to cause adverse consequences (unnecessary restriction, fear of relapse) if relapse alerts prompt clinicians to seek additional information directly from the person with psychosis.	Algorithm-generated relapse alerts should prompt a thoughtful interaction with a trusted human. ASM^a^ data may provide useful additional information to help understand and contextualize alerts from the algorithm.
If DRM^b^ is relied on exclusively, false negatives may result in wasted opportunities for intervention, a false sense of security, and delayed help seeking.	Use DRM as a component of treatment, alongside usual care, not as a replacement.
Where they disagree, it may not always be clear whether the DRM relapse alert, or the information provided by the person with psychosis, is correct.	Clinical skill will be required to weigh up information from disparate sources (the DRM system, the individual with psychosis, wider context) and work out whether the DRM system has produced a true positive (the individual is relapsing but unaware or unwilling to disclose) or false positive (individual is not relapsing).
Participants questioned who should oversee the system day to day (eg, clinicians, an oversight committee).	Depending on available resources and local workflows, consider what would be the most suitable way of integrating DRM oversight into routine clinical care.
Staff with overstretched existing workloads will find it challenging to manage extra data provided by DRM alerts.	Clear local policies are needed to outline how DRM fits with existing workflows to avoid staff being overwhelmed.
There is ethical or political complexity around who oversees the system and who is ultimately responsible. Participants raised legitimate concerns about risks of concentrating power in the hands of unregulated commercial entities, particularly when algorithms operate opaquely.	Governance and oversight mechanisms must be established to ensure that the development and deployment of algorithm-generated systems are transparent, accountable, and not necessarily solely controlled by private companies.
Standard translational pipelines for making DRM systems available to the public are currently lacking.	Establish clear, standardized translational pathways alongside appropriate regulatory frameworks and quality standards to guide the safe, effective, and ethical implementation of DRM systems in clinical practice.
Some participants would find it helpful to share relapse alerts with a family member, caregiver, or trusted other. However, fully informed (and potentially ongoing) consent is essential before sharing DRM-generated data with these individuals.	Robust systems should be developed for regular review of data sharing permissions for family and significant others. Service users should be able to proactively change permission settings when needed. Consider safeguarding implications if service users grant sharing permissions and if they change these permissions.
Participants highlighted factors to consider when choosing a suitable person to share alerts with: geographical proximity, whether the relationship is trusting and supportive, and potential impacts of receiving alerts on the relationship and on the alert recipient themself.	Support DRM users to carefully consider who they wish to share relapse alerts with (family, carer, or significant other). Consider the impacts of receiving relapse alerts on the relationship (will it strain the relationship?) and on the person receiving alerts (will they find it upsetting to receive alerts?). Revisit the decision periodically, considering whether it is still useful to share alerts with that individual.
If a family member, carer, or significant other will receive alerts, first they need information about how to use the DRM system, actions if an alert is triggered, and likely accuracy.	When implementing a DRM system that sends relapse alerts to non-clinical contacts, the implementation plan must include training or information for that individual.

a ASM: active symptom monitoring.

b DRM: digital remote monitoring.

**Table 3.** Trust, fears, and choice: recommendations based on the study findings.

Finding	Recommendation
The patient-clinician relationship is a key factor in whether individuals consent to using DRM^a^ and sharing DRM alerts. Mutual trust and a collaborative clinical relationship are necessary to manage false positives from a DRM system. Authoritative approaches and prior experiences of coercive or restrictive care increased concerns about DRM.	Recognize the power dynamics between clinicians and people with psychosis when implementing DRM systems. Given clinicians’ authority to initiate hospitalization or medication, using algorithm-driven tools in this context requires careful consideration. Safeguards are needed to protect service user autonomy and prevent over-reliance on alerts or other potential misuse.
Using DRM use must be optional.	Ensure that DRM users undergo fully informed consent and have a free choice about whether to use DRM.
Sharing DRM relapse alerts with clinicians must be optional.	Ensure that DRM users undergo fully informed consent and have a free choice about whether to share DRM alerts. Avoid using DRM to replicate existing patterns of coercion.
Allowing users to tailor how and whether they receive relapse alerts themselves may enhance engagement and reduce distress. Some participants preferred not to receive alerts directly, finding them intrusive or anxiety-provoking. Others saw direct alerts as a helpful prompt to monitor their mental health, particularly those living alone or prone to rapid relapse.	In DRM design, build in flexibility to accommodate individual preferences. During onboarding, check whether the individual prefers to receive relapse alerts directly or for alerts to be filtered via a clinician, family member, or trusted other; review this periodically. Consider individual circumstances that may make alerts especially useful. Carefully phrase any relapse alerts that are sent to the user directly to avoid triggering unnecessary anxiety.
Participants acknowledged benefits of algorithm-assisted relapse prediction such as facilitating early intervention, triaging according to need, minimizing human bias, and efficiency in analyzing large volumes of DRM data.	Clinicians should inform service users of potential benefits of DRM and support them in weighing these benefits against possible adverse effects. Discussions about DRM should be embedded within a framework that prioritizes autonomy, choice, and collaborative decision-making.
People with psychosis may be worried about the clinical team overreacting (enforcing hospitalization or medication) or underreacting (not providing additional support) to relapse alerts.	Expectations around what clinicians will do in response to relapse alerts should be clearly articulated before using DRM (during consent and onboarding). Service users may wish to co-create a formal advanced directive stating what they wish to happen if a relapse alert occurs.
Fears of overreaction are not specific to digital tools but reflect broader concerns associated with being a service user in mental health settings.	Recognize that fears of overreaction to relapse alerts are shaped by broader experiences of power and control in mental health services. DRM systems should be used in ways that minimize perceived risk of coercion and actively support collaborative, trust-based care.
Fear of relapse was prominent across themes. Participants suggested that algorithm errors (false positives/negatives) and unsuitable clinician reactions (over/underreaction) may prompt fear of relapse. Fear of relapse may reduce DRM users’ willingness to share relapse alerts with others.	Be aware that fear of relapse is an important psychological factor impacting uptake of algorithm-assisted DRM. Provide reassurance and support to participants expressing fear of relapse. As a previous RCT^b^ showed [43], using DRM systems monitoring relapse risk can reduce fear of relapse in the medium term if suitably supported.
People with psychosis may be concerned that using DRM could disrupt their carefully built therapeutic relationship. In some cases, individuals may prefer to withhold certain information, and the use of continuous monitoring could be perceived as undermining their sense of control or trust.	Consider potential impacts of DRM on the therapeutic relationship, particularly that continuous monitoring may be perceived as intrusive or undermining trust. Respect individuals’ preferences, including their right to withhold information, and prioritize voluntary open communication to preserve a sense of autonomy and mutual respect.

a DRM: digital remote monitoring.

b RCT: randomized controlled trial.

Participants emphasized the need for transparency about algorithm accuracy, the value of a human-in-the-loop approach, and the importance of mutual trust between the DRM user and recipients of relapse alerts. In particular, the inherently ambiguous nature of psychosis symptoms drove participants’ desire for human oversight of DRM. Because understanding symptom changes is contextual and coconstructed, participants emphasized the importance of using DRM with a trusted clinician who understands their communication patterns and can work with them in interpreting subtle changes. Without such trust and understanding, participants feared that clinicians would over- or underreact to DRM relapse alerts. Although participants saw choice about using algorithm-facilitated DRM (and sharing alerts) as essential, they recognized specific benefits of using such a system. To date, this is the most in-depth study to examine the views of people with psychosis on this topic. Nevertheless, findings from existing studies exploring the views of clinicians and people with psychosis on related topics touch on some similar themes.

### Comparison With Previous Work

*Accuracy* of the DRM algorithm emerged as a prominent theme. Although willing to tolerate occasional inaccuracies during early deployment, participants emphasized that limitations should be communicated transparently to users, both initially and when new errors arise. Clinicians also identify accuracy as a key concern [27,29], warning against the use of untested, algorithm-driven digital tools [29] and emphasizing the importance of critically assessing the evidence before implementation [27]. Similarly, mirroring our participants’ concerns, clinicians [26-28] cautioned that inaccurate relapse alerts may cause stress, exacerbate users’ symptoms (eg, anxiety, fear of relapse, and paranoia), waste clinicians’ time, and ultimately cause users to disengage from DRM [26]. Given that the empirical evidence on predictive validity is still nascent and that algorithmic errors may pose a risk of iatrogenic harm, particularly when implemented at scale [44], these are crucial concerns. Indeed, systematic reviews evaluating ML-generated algorithms’ predictive accuracy for relevant outcomes (psychotic relapse [45], schizophrenia treatment outcomes [46], and psychosis prognosis [47]) indicate only moderate accuracy, to date, in studies using wearables [45] or clinical features [46] as predictors; wide CIs [47] indicate high variability across studies. Examples from physical health care (eg, IBM’s Watson, which recommended unsafe cancer treatments) [44] highlight the need for adequate testing and rigorous clinical validation of predictive algorithms before implementation. For psychosis relapse prediction, studies in large representative clinical samples [48], using robust methodology (eg, external validation in independent samples [49,50]), and clearly reporting methods and analyses [51,52] are urgently needed to ensure the safety, reliability, and clinical utility of algorithm-based tools before they are integrated into routine mental health care.

The need for a *human-in-the-loop* approach to algorithm-facilitated DRM emerged as a direct corollary of concerns around accuracy. Participants hypothesized that the potential adverse consequences of false positives (unnecessary restriction and fear of relapse) and false negatives (wasted opportunity and false sense of security) may be mitigated by using DRM alerts to prompt human conversation rather than to replace existing care. Similarly, Starke and colleagues [48] suggested a model of “shared responsibility,” wherein human agents verify algorithmic outputs to ensure that errors do not violate the principle of nonmaleficence (“do no harm”). A key reason for incorporating human input is to provide contextual understanding of digitally gathered data, particularly passive sensing data. Without context, our participants hypothesized that noncritical changes could be unnecessarily escalated, leading to feelings of surveillance or scrutiny. Clinicians report analogous concerns that decontextualized data may cause misunderstandings [26,28] and a clear consensus that data from AI-facilitated tools should be viewed as supplementary information, to be reviewed by a human [26-29]. Likewise, studies from the broader digital health literature repeatedly emphasize that digital tools should not replace human care [16]. Nevertheless, like our participants, clinicians [26-29] worried that DRM could add to already high workloads. Clinicians expressed feeling overwhelmed by existing demands and questioned how DRM could be integrated into clinical workflows without additional resources [28,29]. They emphasized the time needed to learn to use the DRM system, facilitate patient access, understand DRM data, and respond to alerts [26-28]. Implementation science frameworks (eg, Consolidated Framework for Implementation Research) [53] echo this, highlighting that compatibility with existing workflows is a prerequisite for successful implementation [16].

*Mutual trust* between the DRM user and the human-in-the-loop was considered crucial by our participants, previous participants with psychosis [30], and clinicians in a comparable study [26]. Without such trust, participants with psychosis feared an inappropriate response to algorithm-generated relapse alerts—either an overreaction (hospitalization or excessive medication) or an underreaction (no additional support). Such responses may be more likely in overstretched services, where the focus is on managing immediate risks and less time is available to build trusting therapeutic relationships and respond early to signs of deterioration. Staff in previous studies [26,27] admitted they might sometimes distrust DRM information, anticipating that people with psychosis may overreport symptoms (to access support) or underreport (to avoid intervention) and that algorithms may not distinguish genuine from dishonest entries [26]. This contrast between patient and staff views illuminates the importance of mutual trust, particularly when ambiguities arise because the DRM system and the person with psychosis disagree. We observed an interplay between participants’ anticipated trust in the DRM system (related to its accuracy) and trust in the clinical team—if the DRM was highly accurate, trust in the team was less important, whereas a less accurate DRM would require more trust in the team.

Using predictive algorithms in *secondary care mental health services* presents unique challenges. Our findings highlight inherent power imbalances in this setting. Some participants described experiences of paternalistic and coercive care, understandably impacting their willingness to share relapse alerts with clinicians. Echoing previous study participants [30], they feared that clinicians would suddenly invoke their legal or clinical authority to escalate care. One participant cautioned that DRM algorithms may reproduce or exacerbate coercive practices, drawing a parallel with compulsory antipsychotic medication use. The medical ethics literature highlights similar concerns; McDougall [54] warns that “use of AI in treatment decisions can lead to a new form of paternalism, ‘computer knows best’” [55], which could become problematic unless carefully managed. Notably, clinicians [26-29] rarely mentioned power imbalances in mental health services, except in relation to whether DRM should be mandatory. Mirroring our findings, clinician studies unanimously emphasize consent and individual choice regarding DRM use, agreeing that mandatory DRM would be restrictive, coercive, and likely to undermine trust and unfairly exclude people from using services [26]. Clinicians [26] and ethicists [55] advise that all users would need a clearly worded lay explanation of the DRM system. Our participants suggested including information about the algorithm’s accuracy within this explanation, and details of procedures for managing inaccurate predictions. Nevertheless, the opaque nature of many algorithmic predictions makes it challenging to provide a full account of how the system works [44]. Moreover, not all individuals may have the necessary understanding of the technology to provide fully informed consent [26,48,56].

Questions of *responsibility and accountability* were discussed in relation to the DRM system oversight. Similar to our finding, clinicians in a previous study queried who is at fault if the AI gets it wrong—the clinician themselves, their institution, or the AI developer [29]. Issues of personal liability are a pressing concern for clinicians in relation to responding to DRM relapse alerts [26,28] and the broader use of AI systems [29]. To mitigate such concerns, staff urge clear organizational guidelines and policies regarding responsibility for AI-facilitated systems [26,27]. Despite a clear consensus that human oversight of DRM systems is essential, norms around who should provide such oversight are not yet established. Our participants noted ethical complexities, including the legitimate concern that overseeing algorithm-based systems related to others’ health could place individuals in positions of power that might be open to abuse. This raises questions about whether commercial AI developers should hold such power, especially given their financial stake in the systems they design. It mirrors previous patient and clinician participants’ priorities regarding transparency of data processing within digital health systems [57]. Concerns about responsibility also extended to the integration of DRM data into electronic health records. Although clinicians note that automatic integration could facilitate DRM implementation [26], unlike our participants, they did not highlight the importance of ensuring that such records are accurate, in line with data protection principles [58]. If DRM data were automatically transferred to electronic health records without being flagged as potentially inaccurate, future clinicians might interpret it as factual, potentially privileging algorithmic outputs over the patient’s own account. This is a salient concern for individuals with psychosis, who may already feel disbelieved by clinicians. Together, these concerns point to the urgent need for regulation and standards governing algorithm-enabled digital health tools to ensure that these are responsibly overseen and that adverse effects are appropriately managed [59-61].

People with psychosis in this study, along with clinicians in previous studies [27,28], acknowledged *benefits of algorithm-assisted health systems*—efficiency for analyzing large amounts of data, identifying patterns in symptoms or behavior, allowing timely risk stratification, spotting early signs of relapse, and facilitating help-seeking [27,28]. This method may be particularly valuable for real-time analysis of passively sensed data. Our previous paper [25] found that people with psychosis often felt uncomfortable about sharing large volumes of passively sensed data with clinicians. Similarly, other literature reports clinicians’ concerns about receiving too much insufficiently summarized passive data [62]. Generating relapse alerts from passively sensed data may be less intrusive and more useful [25,62]. Overall, these perceived advantages suggest that, when implemented thoughtfully and ethically, DRM systems could enhance care delivery and support proactive intervention. Starke and colleagues [48] argue that “categorical rejection of the use of ML in psychiatry would be ethically wrong given its potential benefits.” This perspective reinforces the importance of balancing caution with openness to innovation, ensuring that the development and deployment of DRM systems are guided by robust evidence, ethical principles, and meaningful input from a variety of stakeholders, including people with lived experience of psychosis and clinicians.

### Strengths and Limitations

This study’s methodological strengths included its large, diverse sample, detailed topic guide, rigorous analysis, and extensive lived experience involvement. Nevertheless, specific limitations may restrict the generalizability of our findings. First, only English-speaking participants were interviewed. Second, despite purposive sampling for diverse characteristics and experiences, people with a specific interest in DRM may have been more likely to participate. Third, although most participants seemed to understand the key principles of ML (after hearing the lay descriptions provided in the topic guide), a minority did not appear to have fully understood these concepts (despite additional explanation and examples), restricting their ability to provide an informed opinion. This may restrict the generalizability of our findings. However, it also highlights that, if ML methods are to be used in clinical practice, it will be important to tailor lay explanations of these methods to individuals’ needs, including the subset of people with psychosis who experience difficulties in abstract thinking [63]. Finally, the timing of the interviews (2022‐2023) should be considered when interpreting the findings, as familiarity with AI has increased over time [64]. Participants may have been less familiar with AI methods than members of the public today, potentially impacting their understanding of and trust in algorithm-assisted systems.

### Conclusion

While participants were generally open to using algorithm-assisted DRM, they stressed the importance of transparency about its limitations and the need for human oversight to mitigate risks of false alerts. Our findings highlight the importance of integrating DRM within a trusted, collaborative care model, ensuring that clinicians are prepared to interpret alerts thoughtfully, maintain open communication, and uphold accountability in decision-making.

## Supplementary material

10.2196/86753 Multimedia Appendix 1 Supplementary information, including topic guide, supplementary methods, sample characteristics, and coding tree outlining themes, subthemes, and codes, with supporting quotations.

10.2196/86753 Checklist 1 COREQ checklist.

## Abbreviations

AI: artificial intelligence
ASM: active symptom monitoring
COREQ: Consolidated Criteria for Reporting Qualitative Research
DRM: digital remote monitoring
*ICD-10*: *International Classification of Diseases, Tenth Revision*
LEAP: Lived Experience Advisory Panel
ML: machine learning
NHS: National Health Service
