# Supplementary material for: Views of People With Psychosis About Algorithm-Based Relapse Prediction and Data Sharing: Qualitative Study
Source: J Med Internet Res. 2026 Apr 10;28:e86753. doi: 10.2196/86753 (PMC13068192; doi:10.2196/86753)
Supplement: Multimedia Appendix 1 [file jmir-v28-e86753-s001.docx]

**Views of people with psychosis about algorithm-based relapse prediction and data sharing: a qualitative study**

Supplementary material

Contents

[S1. Topic guide 2](#_Toc226448198)

[S2. Supplementary methods (reproduced from Eisner et al, 2025) 14](#_Toc226448199)

[S3. Sample characteristics (reproduced from Eisner et al, 2025) 15](#_Toc226448200)

[S4. Coding tree outlining themes, subthemes and codes, with supporting quotations 17](#_Toc226448201)

### S1. Topic guide

**Study title:** A qualitative study of service user and staff views on digital remote monitoring for unusual distressing experiences, psychosis

| **Participant ID number** |  |
| --- | --- |
| **Notes can be made on this topic guide. However, please do not include identifiable information and do dispose of any paper copies securely once the interview has finished.**  **When you finish the interview, you will need to complete a reflective log via Qualtrics**  **Please ensure that this log is completed after each participant and that actions based on the log are taken to the project management group as soon as possible.** | |
| **Prior to interview, research workers should:**  Send participant the consent form, in their preferred format and participant information sheet.  Check they have arranged a time to meet/speak with the participant and, if applicable, that a Zoom, or similar online meeting invite has been sent to the participant and that they have accepted.  The Research Assistant and participant should sit as close to the microphone as possible when using a dictaphone to ensure everything can be easily heard on the recording | |

**Introductions**

- My name is [researcher name]. I’m a researcher on the CONNECT study which is a study about using smartphones and wearable devices, like Fitbits, step trackers or smart watches to help people keep track of their mental health.
- Thank you for agreeing to meet with me today for the research interview. First, I just want to check in about how you are feeling today. Do you feel well enough to do the interview today?
- [If feels well enough, continue] Thank you for reading the participant information sheet and filling in the consent form. Before we get started, do you have questions about anything in the participant information sheet or the consent form? [Answer any questions].

Re-confirm informed consent is still valid and participant still wishes to take part. Key points to cover:

- Interview length: The interview today will last 30-60 minutes.
- Audio recording: We will audio record the interview.
- Anonymity: We will store the interview recording and all other study data securely using an anonymous number rather than your name. It’s best to avoid using full names in the interview, but all names will be removed when the interview is transcribed. We might use quotes from the interview when we publish the research but the quotes will not allow you to be identified.
- Limits of confidentiality: What you say in the interview will remain confidential unless something you say makes me concerned that you or someone else might come to some harm. In that case, I would have to pass the information on to your clinical team or another relevant person.
- Voluntary. It’s your choice to decide whether or not to take part. You can take a break or stop the interview completely at any point without giving a reason, and without penalty.

Are you still happy to take part in the interview?

*[If yes]* Ok, thanks, I’m going to start the recording now ***[Start audio recording]***

Note to research workers: **What follows is a guide.** The order and exact content of the questions will be determined by the participant and will be influenced by the ongoing analysis, so the order of the questions may vary as the interview develops. Prompt and ask for examples as time permits.

Explanations of key concepts and the main interview questions are in blue. Follow-up questions to prompt further discussion are included *in italics*.

| **Topic** | **Interview script/questions/prompts** | **Notes** |
| --- | --- | --- |
| **Overview** | |  |
| Overview of interview | In this interview, I’d like to explore what you think about using a smartphone or a wearable device to help manage your mental health. A wearable device is something like a smartwatch, a Fitbit or a step tracker. Here are some examples [Show Figure S4a].  I’ll start out with some questions on what you think about this topic in general, and then go into some more specifics. Does that sound ok? | Figure S4a: Example smartphones and wearable devices  Figures are provided at the end of the topic guide. Depending on the interview format, the figures can be:   - Printed on card to show to participants - Shared on the interviewer’s screen, if using an online meeting platform |
| Warm up Qs to set the scene and help the researcher know how to pitch later questions | Do you use a smartphone or a wearable device like a smartwatch, Fitbit or step tracker at the moment?  [If yes] What do you tend to use it/them for? *How often?, Daily? Less?*  What is your general view about smartphones and wearable devices like Fitbits, step-trackers or smartwatches?  *How do you find using them? Do you like them? Dislike them?* [If yes, follow up with an open question. E.g. What do you dislike about them? Can you tell me more?]  *Are there things that stop you from using devices like smartphones or wearables?*  *[If yes] Can you tell me a bit about those reasons?* | Note on personalising the interview:   - Briefly ask the participant what word they’d use to refer to a wearable – e.g. “wearable”, “wearable device”, “wearable gadget”, “wearable item”, Then use their preferred wording throughout the interview. - If the participant mentions a specific wearable, you can use that specific example throughout the interview, as applicable. If they use more than one device, make sure you ask questions about each device, as applicable |
| What are the barriers/facilitators to service users using digital tools for healthcare? | What do you think of the idea of using devices like smartphones or wearables for health purposes? That could be for physical health, fitness, or mental health…  Some people might use them for things like counting steps, helping with their sleep routine, setting medication reminders or ordering prescriptions. There are also special devices that people can wear to track things like their blood sugar or heart rhythm.  [If uses devices] Do you use your [name the device(s] for health purposes? Why/why not?  [If uses devices for health purposes] Do you find this helpful/unhelpful? *Can you tell me more?*  *Is there anything that gets in the way of you using technology like this for health purposes? What helps?* |  |
| **Symptom monitoring** | |  |
| Description of symptom monitoring | Smartphones can be used to keep track of your symptoms or how you’ve been feeling day-to-day by prompting you with questions.  I’ve got an example of an app that does this. It’s called the ClinTouch app. [Show Figure S4b]  The app asks you about your mood and experiences, like whether you’ve been feeling low, and whether you’ve been bothered by voices. It can also ask you about where you are and the kinds of people that you’re with, family members, friends, etc, to check out whether certain situations, places or people change how you’re feeling. | Figure S4b: Example symptom monitoring app question, ClinTouch app |
| What are service users’ concerns about active symptom monitoring and what do they perceive the benefits to be? | How would you feel about using an app to keep track of your mental health in this way?  *How would you feel about answering questions on an app about your mood or how you’ve been feeling? What would you find helpful/unhelpful?*  *How would you feel about answering questions about unusual distressing experiences like hearing voices or feeling very suspicious?*  *How would you feel about answering questions about where you are and who you’re with?, To see if any situations, people or places influence your symptoms.*  *Would anything worry you about using an app to keep track of your symptoms or feelings in this way? [If yes, what in particular?]* |  |
| How can we keep service users engaged with symptom monitoring? | How would you feel about tracking your mental health in this way long term? For example, for 12 months?  What would help you *keep on* tracking your mental health like this long term?  *Is there anything we could build into the app to help keep you engaged?* |  |
| Views on sharing information with clinical team | If you were to track your mental health using an app like this, your answers could be sent electronically to your mental health team: this could let them know if you need any extra support, for example, an extra visit or a doctor’s appointment.  How would you feel about this? Would you find this helpful/unhelpful? Why? *Can you tell me more about that?* |  |
| **Passive sensing** | |  |
| Description of passive sensing | We are going to move on to talk about passive sensing now. Smartphones and wearables, like Fitbits or smartwatches can gather lots of continuous information, without you having to *do* anything apart from wear the wearable or carry your phone around with you. This is called passive sensing. Here are some examples [show Figure S4c and give the participant some time to look at the examples of info tech can gather].  This information tells a story about the kind of things you’ve been doing – like whether you’ve been sleeping, exercising, travelling, or phoning people.  In turn, this can give clues about how you might be feeling. For example:   - Someone who is feeling very anxious or low might start socialising less. Their smartphone could gather information about their location and phone-use, that shows they are staying at home more and messaging people less, so it could spot that change.   What it can’t tell us, though, is why someone might be staying at home more than usual, or why someone’s sleep might be affected.  So, this process is different to using an app to record your experiences like we talked about before, because this information is collected automatically without you having to put any information into the phone or wearable device yourself.  Do you have any questions about how passive sensing works or anything I have just said? [clarify any uncertainty] | Figure S4c: Types of information gathered by smartphones and wearable devices |
| What are service users’ concerns about passive sensing and what do they perceive the benefits to be? | What do you think about collecting information in this passive away? How would you feel about information like this being collected about you?  *Would you feel comfortable with this? [If yes/no] What in particular might you feel comfortable or uncomfortable about?*  *How might it help? What might the benefits be? Can you see any disadvantages?*  *Do you think your view might change over time – for example, might you feel differently about this on a day when your mental health was better or worse than today?* | Note: If they seem unsure or say they are not sure, clarify what the specific uncertainty is and whether they need more explanation about what passive sensing does and how it works |
|  | The information collected by the smartphone or wearable could be sent to someone’s mental health team too, so the team could be updated about changes in someone’s activity. The team could then offer the person extra support.  What do you think about this type of information being shared with people’s teams in this way?  *What would be helpful/unhelpful? Why?* |  |
| What can be done to make passive sensing technology more acceptable to users? | [If they have concerns…] Is there anything that could help you feel more comfortable about health-related information being gathered in this passive way?  [If they don’t have concerns…] Is there anything that you think we could do to help address other people’s concerns about this?  *Would you want to see a copy of the information the smartphone or wearable has gathered? [If yes] How would you want to receive this?, e.g. in the app, via email/post. How often would you want to see this?, E.g. just once, or on an ongoing basis?.*  Smartphones can gather information about where you are throughout the day.   - How would you feel about the app knowing your exact location? - How would you feel about the app knowing the general area that you were in, e.g. [insert relevant local area. E.g. Fallowfield]? - How would you feel about the app knowing how far you’ve travelled from home each day, without knowing where you were travelling exactly to or from? - *Which of these options would you feel most comfortable with?*   I am interested in what kinds of health-related information you would be comfortable with the smartphone or wearable gathering. Let’s look back at the examples we saw earlier [Show Figure S4c and give time to read the examples there]. Do any of these types of information cause you concern? Which ones? Why? |  |
| **Relapse** | |  |
| Description of relapse | People who have mental health problems like psychosis or schizophrenia may experience times when their symptoms worsen and become more problematic. This worsening is sometimes described as a ‘**relapse**’.  We want to help people to manage their mental health and minimise relapses. To do that we want to spot early when people are starting to relapse. That way, people can quickly get the help and support they need.  *[pause and check for understanding so far]*  We’ve talked about two different ways that technology might help people keep track of their mental health: 1 by allowing them to report their symptoms in an app; and 2 through passive sensing, where ongoing health-related information is gathered from the smartphone or wearable without the person having to do anything. In our CONNECT research study, we are looking at whether we can use these methods to spot early that someone might be getting unwell, before this is normally noticeable. |  |
| What are service users’ views on active/passive monitoring for *relapse prediction* specifically? | What do you think about using these methods to spot early signs that your mental health may be getting worse?  What might be helpful about this? What might be unhelpful? *Can you tell me more about that?*  *Would anything worry you about using technology to do this? [if yes] What would you be concerned about?* |  |
| **Machine learning** | |  |
| Description of machine learning | We are now going to move on to talk about a technique called machine learning. I’ll explain what I mean by that…So, using smartphones and wearables in the ways we’ve talked about collects a large amount of health-related information. For that information to be useful, we need a way of making sense of it, analysing it. One way of making sense of it is to use a technique called **machine learning.**  YouTube and Netflix use this technique. They gather lots of information about people’s viewing patterns and then make recommendations to viewers about what they might want to watch next.  In our CONNECT research study, we’ll use smartphones and wearables to collect a lot of health-related information from a lot of people with psychosis. Then we’ll see if we can use machine learning methods to pick up changes in people’s mental health.   - First, we’ll have a training phase where we teach a computer to recognise patterns in the information we’ve collected. For example, one pattern might be that people tend to leave the house less as their mental health is worsening. - In the training phase the computer gets lots of human guidance and supervision. - When the computer has finished its training, it can then spot patterns without human supervision.   Do you have any questions about this so or what I’ve said so far? |  |
| What are service users’ views on machine learning for *relapse prediction* specifically? | How do you feel about this process of a computer program learning to pick up changes in people’s mental health?  *Do you have any comments or concerns about this method? [If yes: how so? Can you tell me a bit more about that? What are your concerns?]*  *Is there anything that would worry you about a computer program picking up when you might be getting unwell? [If yes, what in particular?]*  *How would you see a computer program like this working alongside the usual care that you/other people receive from the mental health team? Would it feel different to the usual support on offer? Why/why not?* | Note: If they seem unsure or say they are not sure, clarify what the specific uncertainty is and whether they need more explanation about what machine learning does and how it works |
| What do people think about this information being shared with others? | If the computer picks up that someone’s mental health may be worsening, this information could automatically be sent a person’s care team. How would you feel about this? *What would be unhelpful/unhelpful about this?*  How would you want your care team respond to seeing an alert that your mental health is worsening? *What would be helpful/unhelpful? Why?*  How would you feel about an app electronically alerting a parent, carer, trusted other or supporter about those changes with your permission? *How would you want them to respond to this alert? What would be unhelpful/unhelpful? Why?*  How would you feel about an app alerting you directly? *What would be helpful/unhelpful about this? Why?*  *In general, how would you want to see this information?, e.g. in app alert, email, text* |  |
| What concerns do service users have around machine learning systems/ algorithms? | *What would you need to know to feel comfortable about services using this method as part of your healthcare?*  *What would we need to consider, from your point of view, to make this an acceptable way for services to support you? What could make things easier?*  *Is there anything we could do to address people’s concerns about using this method?* |  |
| What proportion of false negatives and false positives is acceptable for predicting relapse? | Just like people get things wrong sometimes, so can computer programs. The program won’t *always* get it right. Like with your care team, the computer might think your mental health is getting worse when actually it isn’t…and it might sometimes miss when you are becoming unwell.  What do you think about the idea that the program won’t *always* get things right?  *Do you have any concerns about this? Can you tell me more about that? Would this affect your willingness to use technology in the ways we’ve talked about?*  *Which would be worse for you – if the computer said your mental health was getting worse when you were actually fine…or if the computer didn’t pick up that you were actually getting unwell? Why is that?*  *How should we tell people that the computer got it wrong? How can we manage this?*  *(What do you think about the idea that your doctor gets things wrong?* |  |
|  | |  |
|  | In future, a system that uses smartphones or wearable devices to help people keep track of their mental health might eventually become part of the standard care offered by mental health teams, so that everyone under the care of a mental health team would use a smartphone/wearable system in the ways we have talked about.  What do you think about this?  Do you think should people be able to opt out of using a system like this? Why/Why not? | [Note: this is intentionally controversial to get participants thinking about what they would or would not be happy with] |
| **CONNECT** | |  |
| Consent procedures  What concerns do service users’ have around data sharing and trust online? | What would you want to know before you agreed to track your mental health using a smartphone/wearable?, *e.g. storage location/duration, who has access*  *If someone was going to start using a computer program like this as part of their mental health care…what do we need to tell them?*  *How much detail would you want to know about how the information was being stored and used?*  How often would you want to be reminded that information was being gathered from your smartphone or wearable in order to check that you still agree to this?, *e.g., Monthly? Every 3 months? Every 6 months?* |  |
| Views on ethical/ governance issues associated with data tracking/storage in a digital health context | What could we do to help you trust this kind of system?  What do you think we could do to help you feel confident that your information is being stored safely and securely?  *Who should have access the smartphone or wearable information?* |  |
| Is the proposed digital health data management plan for the CONNECT study appropriate and acceptable? | In our CONNECT research study we will collect information from smartphones and wearables to use in the ways we’ve discussed today. We will store that information for at least 10 years in a safe, password protected folder on a university or NHS computer system.  How does this sound to you?  *Does this sound safe? Acceptable? Secure? Is there anything else we should be considering? Is there another way we could do this? [If yes, How?]*  Would you be happy for other researchers around the world to have access to anonymous information to answer important scientific questions?  [If no] What are your concerns about it? |  |

Interview closedown

- Is there anything else that you would like to tell me that we haven’t discussed?
- How have you found this interview today? How are you feeling now that we’ve done the interview?
- How do you think this interview could be improved for future participants?
- Ok, I'll now switch off the audio recorder ***[Turn off recording]***

Thank you for taking part in the interview. Just before we finish I’ll go through some very quick demographics questions with you if that’s ok – just about your age, employment status etc.

***[Go through demographics Qs and input directly into Qualtrics]***

Finishing

- Thank participant for taking part and let them know that their contribution to this research is extremely useful and important.
- Explain what will happen now and how this information will be used
- Offer to provide summary of the findings when available
- Offer a phone call: Sometimes people take part in an interview and afterwards they have more questions to ask, or they have been worrying about something they said. If you like, I can call you tomorrow or the next day just to check if any of this is happening for you – would you like me to do that?

***[Complete reflective log via Qualtrics]***

####
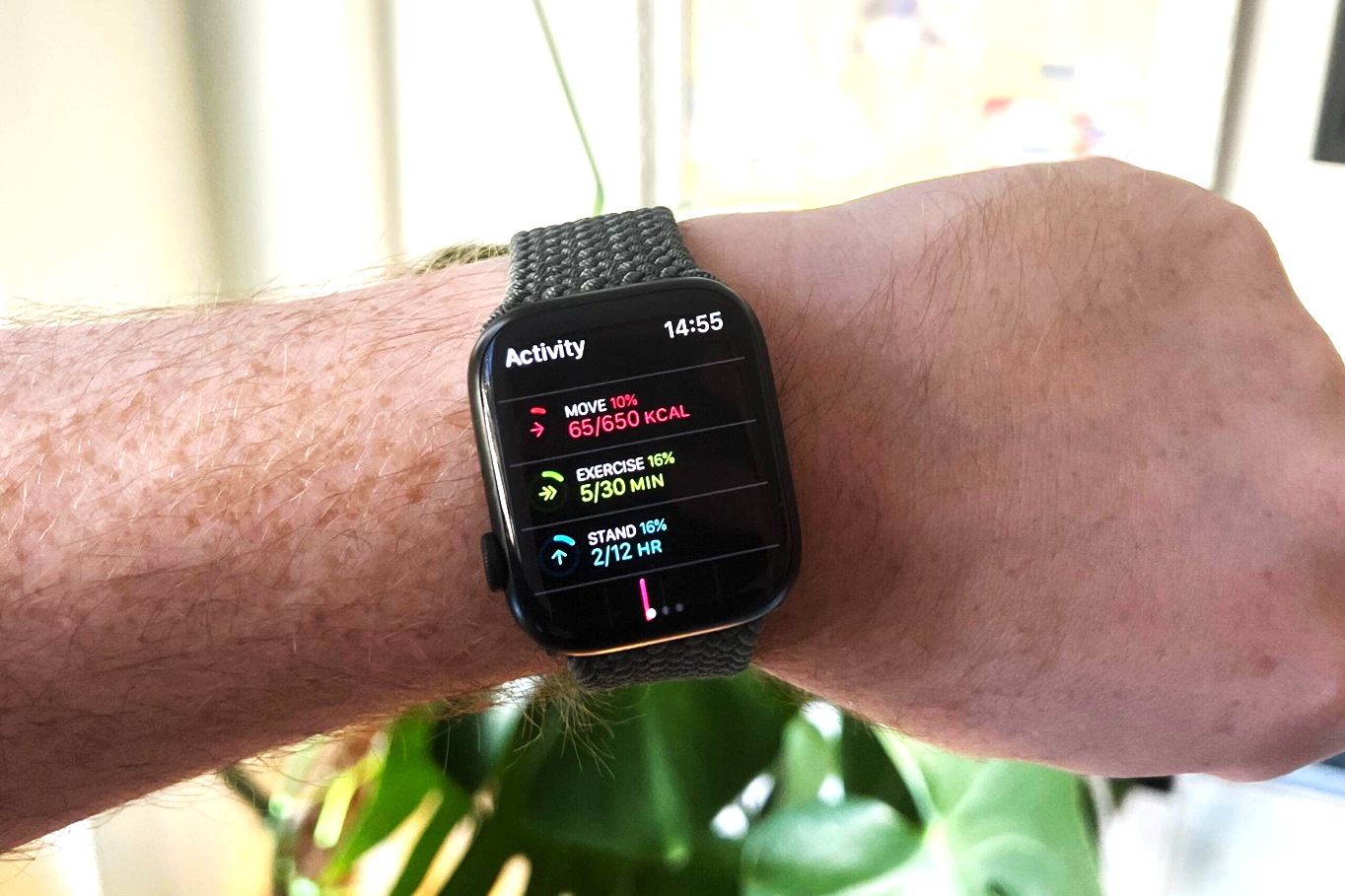

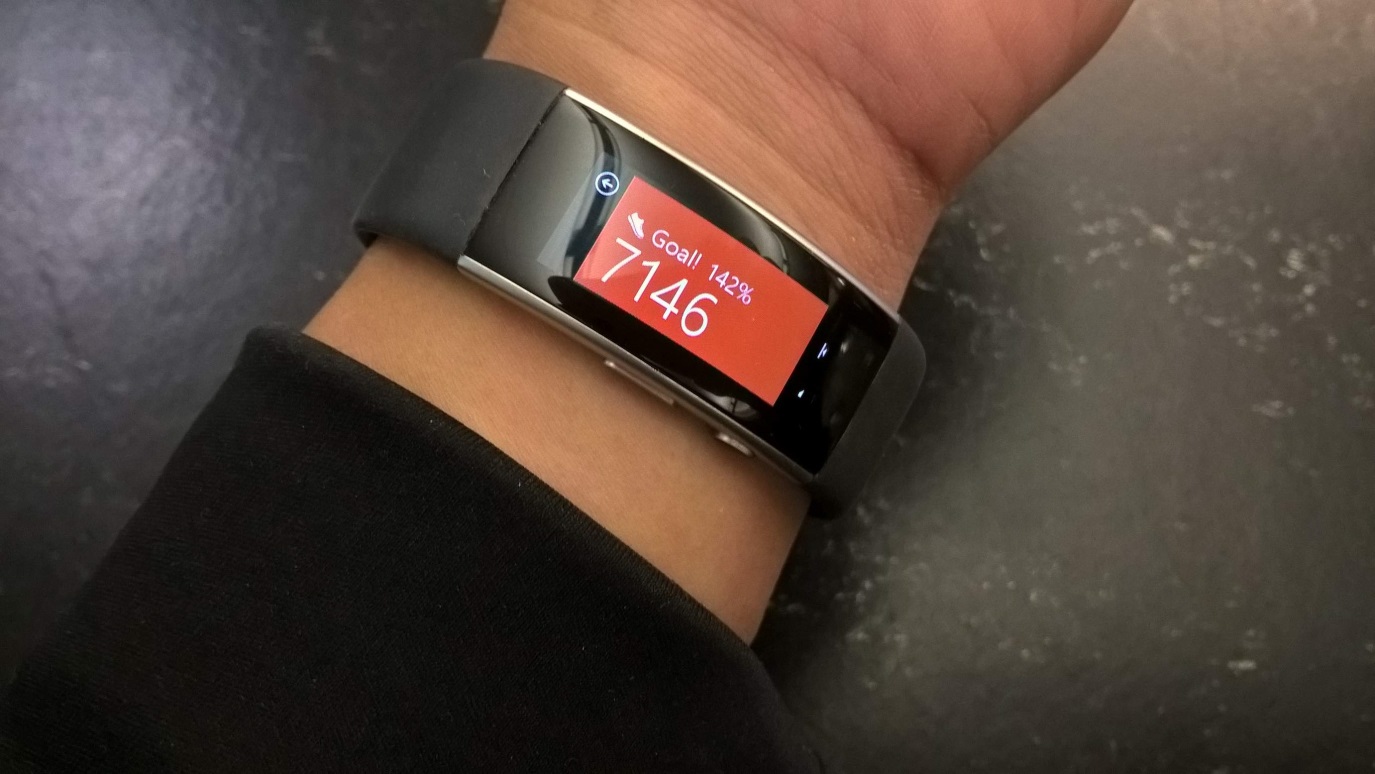
Figure S1a: Example smartphones and wearable devices


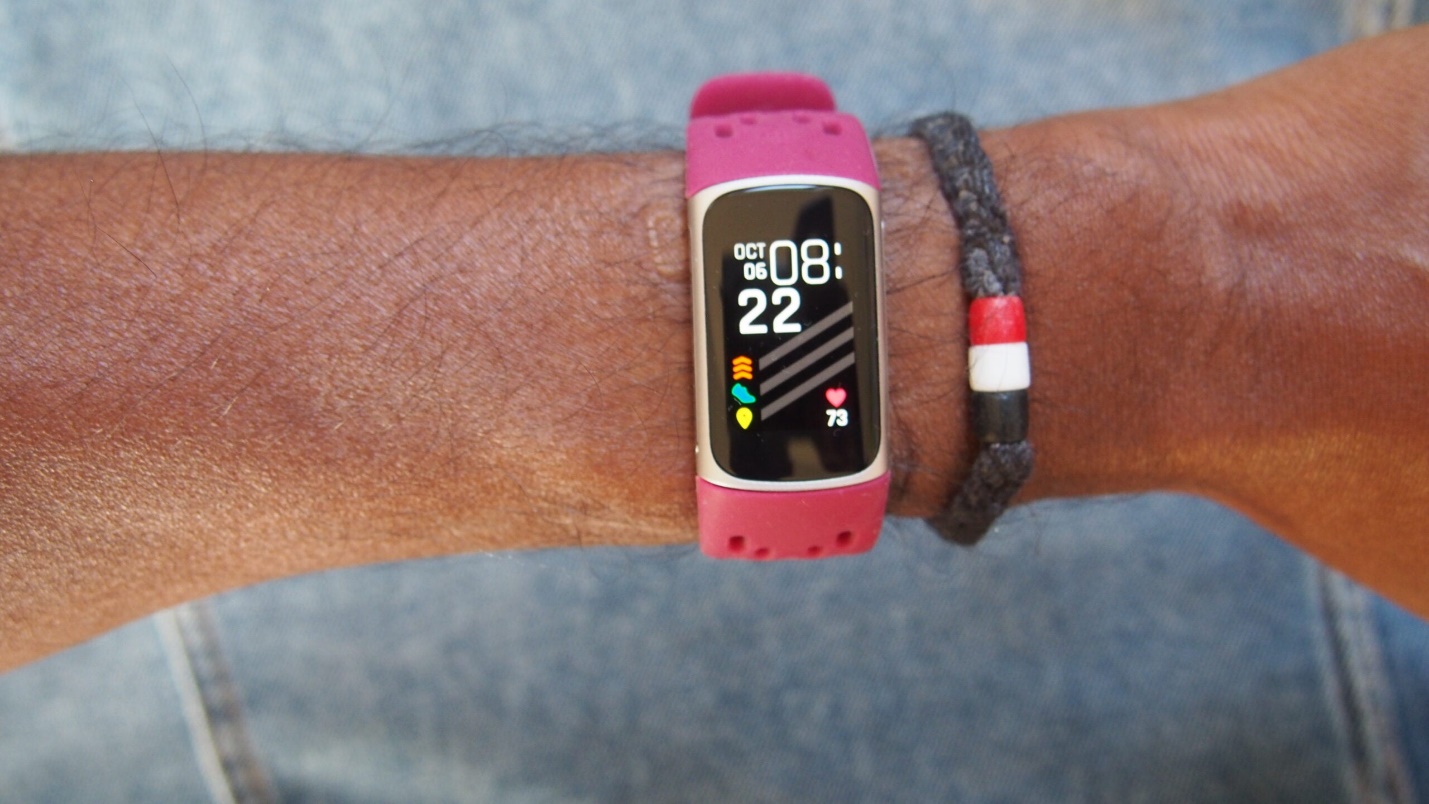

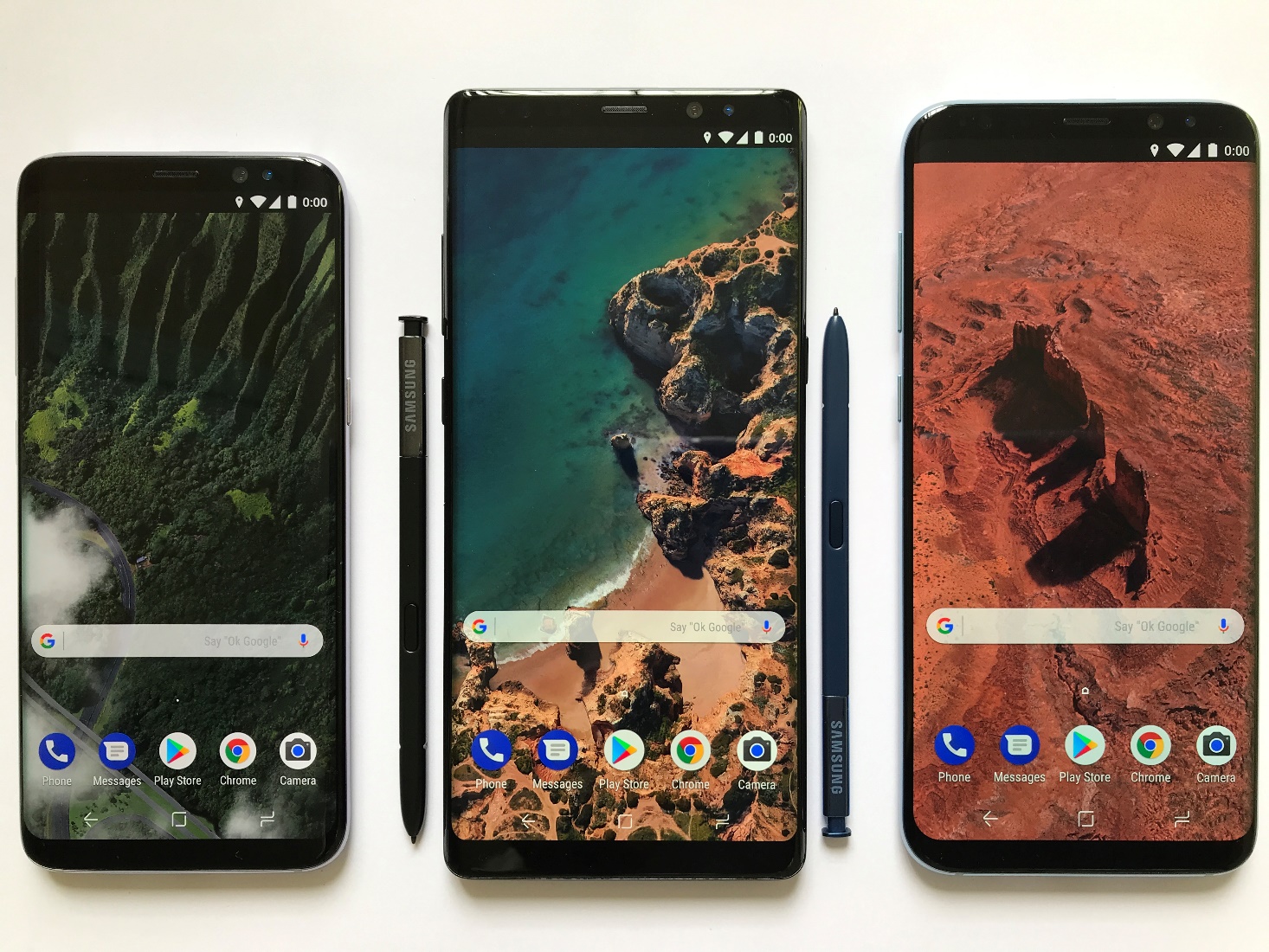

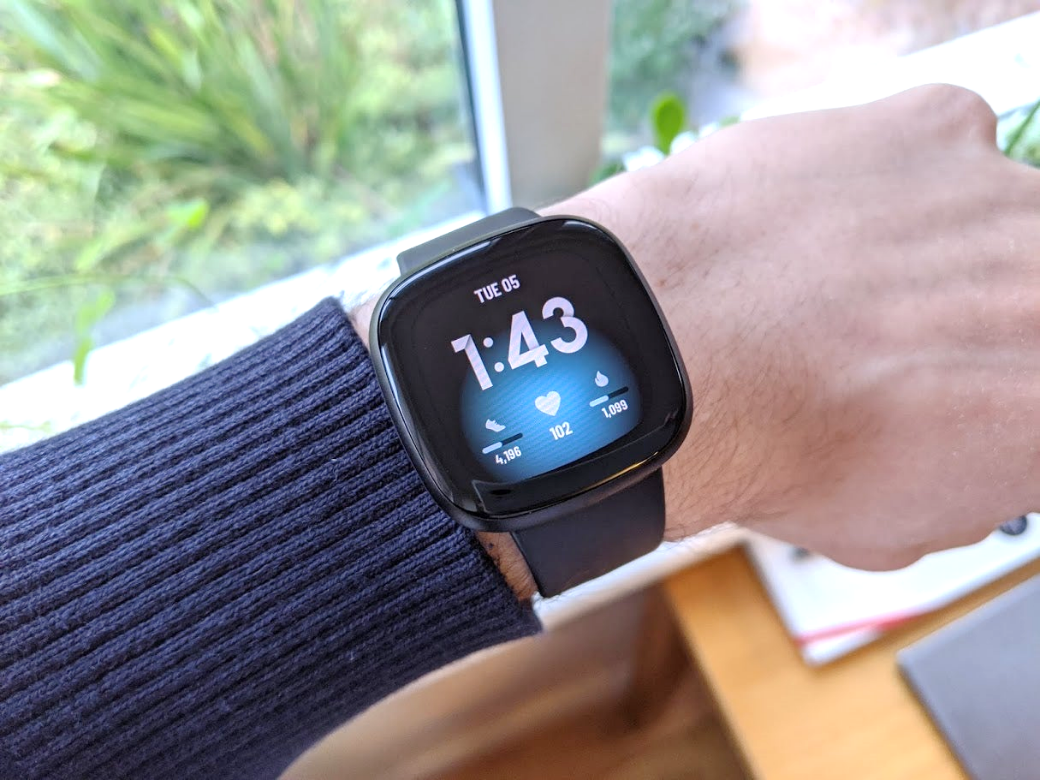

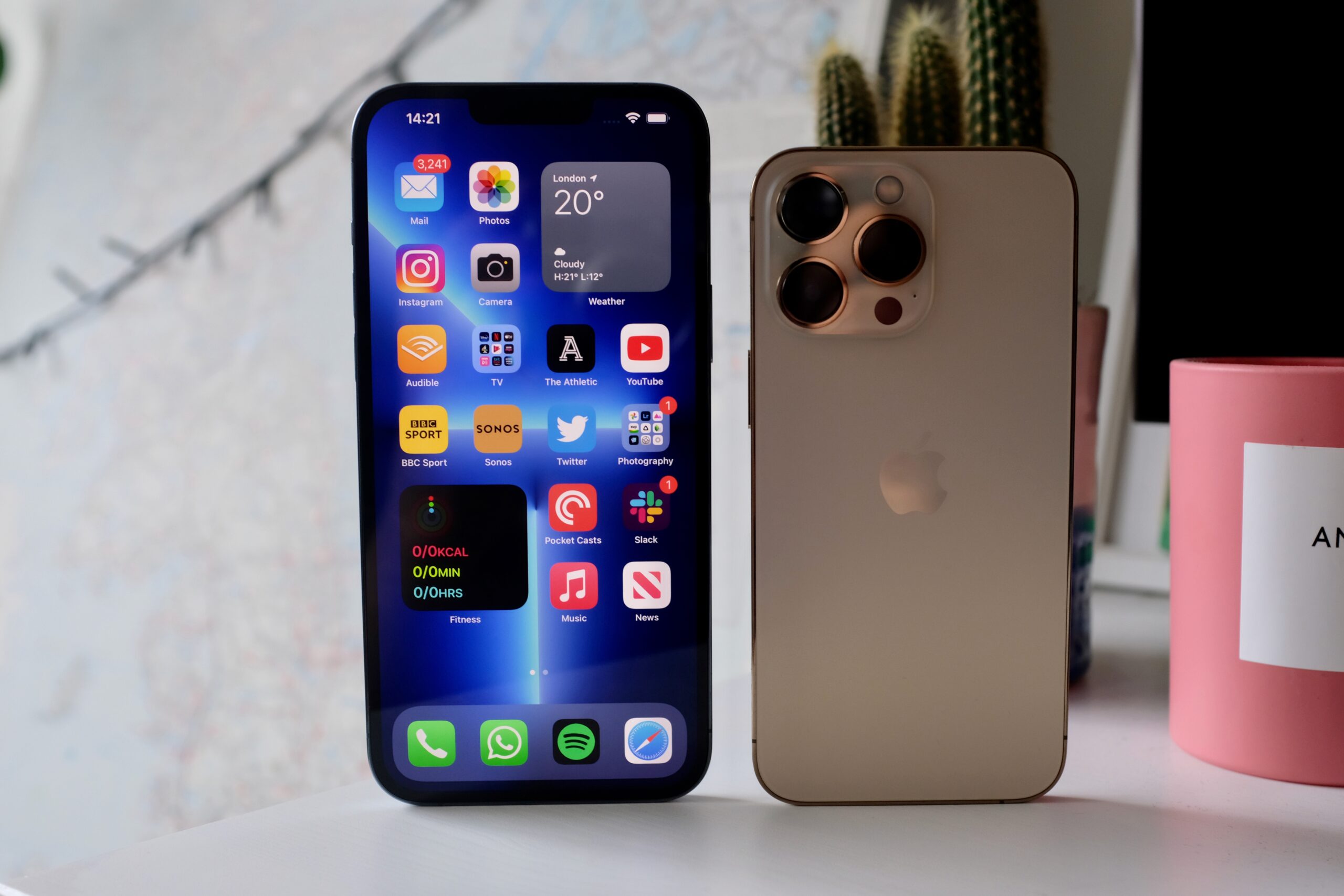


Smart watches Step/fitness trackers

**Smartphones Wearables**

#### Figure S1b: Example symptom monitoring app questions, ClinTouch app


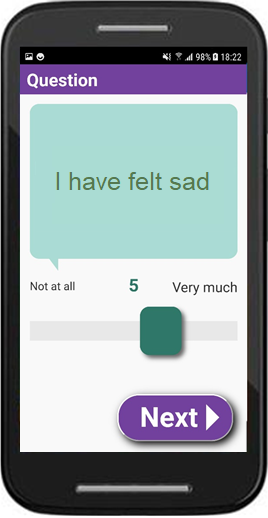


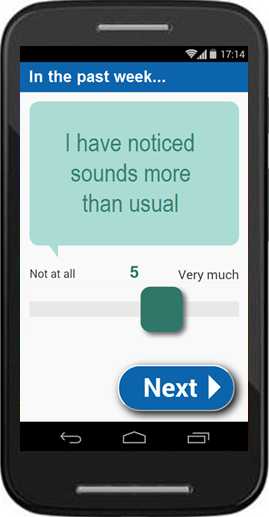

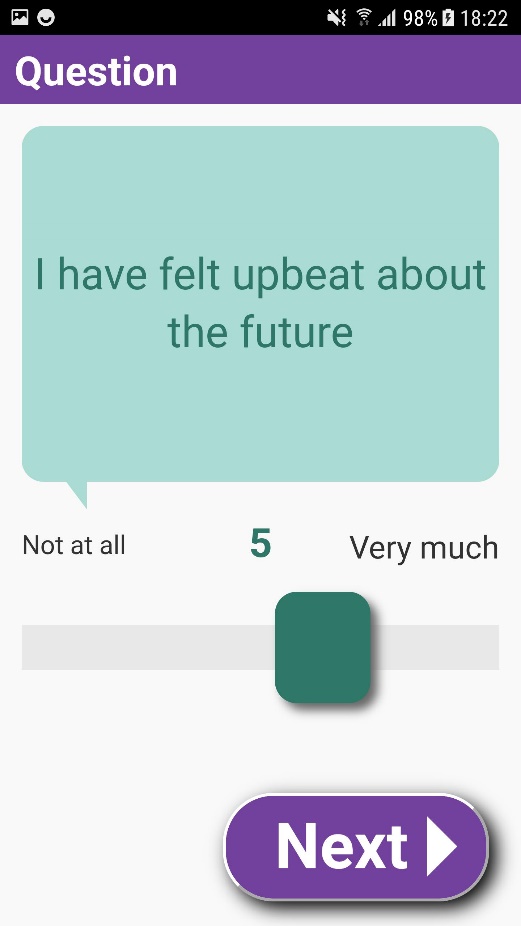

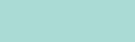


I have heard voices

#### Figure S1c: Types of information that can be gathered by smartphones and wearable devices


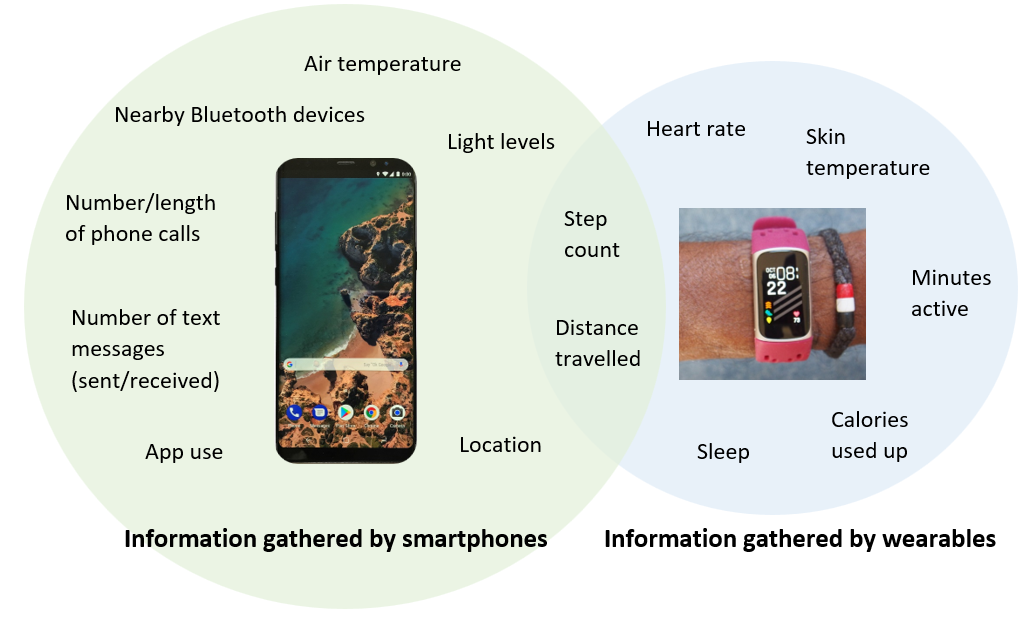


### S2. Supplementary methods (reproduced from Eisner et al, 2025)

Eisner, E., Ball, H., Cella, M., et al. (2025). Using Passive Sensing to Predict Psychosis Relapse: An In-Depth Qualitative Study Exploring Perspectives of People With Psychosis, Schizophrenia Bulletin, sbaf126, <https://doi.org/10.1093/schbul/sbaf126>

#### Epistemological approach

Consistent with a non-positivist epistemological stance, no attempts were made to establish inter-rater reliability. This approach acknowledges and values researcher and lived experience subjectivity as an integral component of the interpretive process, recognising potential bias rather than seeking to control or eliminate it.^29,32^ Data analysis was underpinned by a critical realist epistemological approach, which is particularly well-suited for exploring complex social phenomena such as the implementation of DRM in mental health services. Critical realism assumes that objects, including social objects, exist but that our attempts to describe them are fallible.^33^ This approach acknowledges researcher and participant subjectivity but allows tentative transferability of study findings by aiming to explain and understand events beyond the study sample.^34^ This epistemological stance is especially valuable in the current study, as it facilitates a detailed exploration of participants’ experiences, acknowledging both their subjective interpretations and the broader structural and contextual influences on DRM adoption. For example, participants’ diverse prior experiences of mental health services (e.g., supportive, paternalistic, or coercive) are likely to affect their views on using DRM in this context, as are their experiences of wider issues such as societal stigma.

#### Reflexivity

All researchers were part of the CONNECT study research team which included academics, researchers, clinicians and people with lived experience of psychosis. Researchers who completed the interviews were primarily employed as local site researchers on the study. Interviewers, n=9; CR, HB, KO’H, LM, NC, RT, SC, SF, UZ were all women, and all had relevant doctorate/master’s degrees. Interviewers received study-specific training by SB, HB, and EE, who are experienced in qualitative research methodology. Training covered general background to qualitative research, interviewing skills and role-play interviews with feedback. HB, a female Clinical Psychologist and PhD researcher investigating the implementation of digital technologies in psychosis care, provided supervision to interviewers, which included listening to interview recordings and providing feedback.

Most participants did not have pre-existing relationships with interviewers, but a small minority knew their interviewers in a professional capacity. One participant’s partner was present during the interview to support the participant. Researchers conducting the interviews or analysis had some background knowledge of the topic and likely brought assumptions and biases, e.g., feeling invested in the idea of a DRM system being used in future mental health care. All participants were aware that interviewers were researchers involved in the CONNECT study and thus may have assumed an inherent interest and favourable bias towards using digital technology in mental health care.

#### Eligible individuals declining to participate

Across all sites, only 11 of the 69 eligible individuals who were invited to be interviewed declined to participate. Cited reasons included being keen to "move on with life", being currently unwell, feeling despondent with healthcare, not being interested in using technology to monitor mental health, and scheduling challenges due to job hunting and other commitments

### S3. Sample characteristics (reproduced from Eisner et al, 2025)

Eisner, E., Ball, H., Cella, M., et al. (2025). Using Passive Sensing to Predict Psychosis Relapse: An In-Depth Qualitative Study Exploring Perspectives of People With Psychosis, Schizophrenia Bulletin, sbaf126, <https://doi.org/10.1093/schbul/sbaf126>

|  | **Frequency (n=57) ^a^** | **Percentage** |
| --- | --- | --- |
| Gender ^b^ |  |  |
| Man/male | 32 | 56.1 |
| Woman/female | 25 | 43.9 |
| Ethnicity |  |  |
| Asian/Asian British | 6 | 10.5 |
| Black African | 5 | 8.8 |
| Black British | 4 | 7.0 |
| Mixed ethnic background | 2 | 3.5 |
| White British | 37 | 64.9 |
| Other ethnic background | 3 | 5.3 |
| Relationship status |  |  |
| Co-habiting | 7 | 12.3 |
| Divorced or separated | 2 | 3.5 |
| Married | 2 | 3.5 |
| Partnered | 4 | 7.0 |
| Single | 42 | 73.7 |
| Employment status |  |  |
| Employed | 12 | 21.1 |
| Unemployed (looking for work) | 3 | 5.3 |
| Unemployed (not looking for work) | 5 | 8.8 |
| Retired | 3 | 5.3 |
| Self employed | 6 | 10.5 |
| Student | 3 | 5.3 |
| Unable to work | 19 | 33.3 |
| Voluntary work | 6 | 10.5 |
| Parental or caring responsibilities | 10 | 17.5 |
| Child(ren) | 7 | 12.3 |
| Family member | 1 | 1.8 |
| Parent(s) | 1 | 1.8 |
| Would rather not say | 1 | 1.8 |
| Education |  |  |
| Secondary school (GCSEs) | 14 | 24.6 |
| Further education (6^th^ form, college or  equivalent vocational education) | 21 | 36.8 |
| University Bachelor’s degree | 14 | 24.6 |
| University Master’s degree | 4 | 7.0 |
| PhD or higher | 2 | 3.5 |
| Other education | 1 | 3.5 |
| Living situation |  |  |
| Alone | 24 | 42.1 |
| With parents/carers | 12 | 21.1 |
| With partner | 8 | 14.0 |
| With children | 3 | 5.3 |
| With other relatives | 1 | 1.8 |
| With friends or similar | 6 | 10.5 |
| Supported accommodation or similar | 3 | 5.3 |
| Self-reported diagnosis |  |  |
| Psychosis | 22 | 38.6 |
| Schizophrenia | 26 | 45.6 |
| Schizoaffective | 4 | 7.0 |
| Bipolar | 3 | 5.3 |
| Other | 1 | 1.8 |
| Missing | 1 | 1.8 |
| Current mental health service |  |  |
| Community Mental Health Team | 27 | 47.4 |
| Early Intervention Service | 15 | 26.3 |
| Inpatient | 5 | 8.8 |
| Psychiatry outpatients | 4 | 7.0 |
| Forensic outreach team | 1 | 1.8 |
| Rehab or recovery team | 2 | 3.5 |
| None | 2 | 3.5 |
| Missing | 1 | 1.8 |
| App installed on a device ^c^ | 27 | 47.4 |
| Mental health | 13 | 22.8 |
| Physical Health | 17 | 29.8 |
| Wellbeing | 9 | 15.8 |
| Mood and/or Health Tracking | 7 | 12.3 |
| Other health related app | 3 | 5.3 |
| None | 30 | 52.6 |

^a^ Sample size is 58 but all demographics missing for one participant (M009)

^b^ All participants reported that their gender matched the sex they were assigned at birth

^c^ Participants were asked to report which types of app they currently have installed on their digital device (phone, tablet or wearable). Frequency of app sub-types adds to more than 57 because some people reported more than one type of app.

### S4. Coding tree outlining themes, subthemes and codes, with supporting quotations

| **Theme or**  **subtheme** | **Participant quotations** |  |
| --- | --- | --- |
| **1. Accuracy** | |  |
| 1.1. Learn from initial inaccuracies to build trust in the algorithm | Initial inaccuracies inevitable and understandable   - “I think it’s understandable that it would take time for it to perfect it, so there would obviously be mistakes in the infancy of it”, E007 - “Well it’s just part of life really, isn’t. These things happen”, G006 - “I would expect that, being the beginning of it learning. Yes, I wouldn't mind. But I think there are some people that it might bother”, M003 - “It’s inevitable. It’s sad obviously you want things, want it to get it right all the time but, realistically everyone should know it’s not always gonna get it right”, M011 - “I suppose it’s going to be a hit and miss anyway, not everything, like you say, with mental health I think it’s really…everyone gets something wrong, it’s just, you just have to, it’s just going to be something you’d have to cope with if it comes to it”, M004 - “because yeah…and it's probably a while off yet before, you know, it's completely accurate”, C002 - “it’s a good idea, but erm, there’s I guess there’s questions about how reliable is it, if this is the first time its being used for something like this”, G008 - “Especially if the technology is like quite new…I think it’s going to be a few years before that would be helpful”, C002   Normal for software to have initial teething problems   - “I suppose if any like, um, new Software or or new data management system, there are going to be teething problems”, C005 - “You don’t get everything right…But eventually it will it’s just taking a while, like it took YouTube years to get its algorithm right”, C007   However, errors should be corrected to improve accuracy over time   - “Well it’s a program, it’s in its early stages of working, there is gonna be problems…unless you work on it and iron out the problems…And you can only do that once you start to collect the information…you can’t do that and fix any problems until you’ve got everybody working on the app”, E004 - “I think there’s never going to be a hundred per cent level of certainly but if you’ve got self-learning AI or if you’re doing machine learning, then it’s more likely that those mistakes will be used to correct it”, M008 - “I think it’s gonna be a process of trial and error”, C004 - “Think it’ll probably have to go through some sort of trial period where the people using it are aware that it’s not at its best at the moment”, K004 - “It can't get everything a hundred per cent right. As time goes on, things should get better. If they start developing watches and the ’phones and more people are willing to actually have the ’phones and the watches, obviously…will there be a trial period for a while if they do get it up and running?”, K009   If the algorithm is inaccurate, it would not useful so participants would stop using   - One participant reflected on their reaction to inaccuracies they had experienced with the Fitbit stress algorithm: “I suppose it is obviously very low key with my Fitbit, stress things like that. I have noticed I do just not really engage with it, so I do wonder if it would be similar. If I might just feel, this isn't useful, so maybe stop engaging”, G001 - “If the technology was wasn’t being accurate, it would, yeah, I wouldn’t want to use it”, G004 - “If the programme like makes up or doesn’t catch information, then why are you working with the programme if it doesn’t help?”, K010   Service users and staff will trust the system less if they think it is inaccurate   - “It does matter…It’s like the boy who cried wolf!”, S002 - “This is serious, it’s schizophrenia, people are going into, you know, it’s people’s mental health and it’s not a joke is it”, S002. - “That would stop me from it using yeah, ‘cos that would be a kink in the system. It would be my whole health, mental health as well as my physical health and everything else…if it was to breakdown…. breakdown in communication that would stop me from using…iPhones are a communication device. A breakdown in communication, that would stop me from using it”, E003 - “At some point this will lead to miscommunication or misunderstanding. You know, like an app will not…the app is meant to report how your relapse is or how you’re feeling at the time. I would like to say there would never be an accurate communicative line through any form of apps”, M002 - “It’d put doubt in their head…with people with mental health it’s all about that trust…so instantly that trust is lost”, C007 - “If [clinicians] then get sent this every single day and sometimes it's misinformation as well, they're just probably going to be reluctant to even take note of it…it might come through but they might be like, ‘oh, you know, this programme, it's not worked before, so how can we sort of trust it?’. So I think…trusting the actual technology is really important because yeah…and it's probably a while off yet before, you know, it's completely accurate”, C002 - “Unless I’ve said oh no you’ve got it wrong I’ve been at home all day, you know, for the last three months but you’re saying I was out in Blackpool for three (laughs), you know, I was out of the house for, for a two month holiday and something went wrong and then, and then there’d be that trust issue”, M011 - “it’s a good idea, but erm, there’s I guess there’s questions about how reliable is it, if this is the first time its being used for something like this”, G008 - “Especially if the technology is like quite new…I think it’s going to be a few years before that would be helpful”, C002   Test fully before use by patients.   - “I’d like to think it was, before anything was launched, that’d it be…well tested out first”, G002 - “I’d want to see it being rigorously tested”, K003 - “It needs to be trained trained trained trained trained…I know there’s mistakes made but this is a very serious issue, so it should be checked checked checked”, S002 - Needs to be “fail proof: mistakes cannae happen”, G003 |  |
| 1.2. Transparency about accuracy | DRM users should be informed up-front of the limitations of the DRM system:   - “Maybe even a statement saying it might not be 100% accurate but the app is continuously trying to make improvements”, C004 - “If a support worker was coming to help an individual and they were coming to the individual because of the app and its alerts maybe that the people in healthcare should know that the app is not 100% accurate themselves so that they can spread that information out to individuals and know that there are other resources they can tune in to, and umm they can just provide the resources then and there”, C004 - “A warning saying ‘caution computer may come up with the wrong conclusion’”, E003 - “There are always limitations in any machine learning method…I think anyone who’s participating or using that, needs to be aware of this”, K001 - “I think that should be shared with people using it, that it might not get things right and how to go about dealing with that when it doesn’t get things right”, K004 - “I think you should just describe that you need a lot of patience with the computer as well, just as the way they use their smartphones and the devices at home, sometimes they don’t work properly. So just try and make it like it’s not something that you expect a hundred per cent from. So to have a bit of a leeway to make mistakes as well”, M005 - “Realistically everyone should know it’s not always gonna get it right, and as long as we have that honest conversation and say well in this, you know, if this was to happen then we can, can talk about it…I think yeah, just be honest about it yeah…And that it’s not something that’s gonna go on your record”, M011   Communicate transparently and promptly when errors occur   - “I think be open and honest. You’re either gonna win them or lose them if you know what I mean, in regards to that so the best thing to do is be honest and you know, that’s the best way to approach it really…‘Cus even if they go I don’t want to take part in it anymore, you know, you’ve done the right thing by telling them, oh it was a computer error if you know what I mean”, C007 - “Just be open and honest”, K001 - “It’s just saying there’s been a wee blunt, something’s happened, here’s what’s happened, it shouldn’t have happened but it’s fixable so…It’s fine”, G006 - “Tell them in some way straight away”, M010 - “You should have to say there’s something wrong with the technology and the accuracy of the information reported and eh do some sort of identification about what’s gone wrong…and deal with that”, G004 - “Just be open and honest. And what you’ve got to do in response, if you’re going to, perhaps it’s an indication of a retraining need?”, K001   Check how the DRM user feels about the error and/or take into account their potential feelings   - “Keeping channels open and maybe actively leaning in, how people are finding things”, G001 - “I guess it varies person to person. Someone could find it more erm frustrating I guess”, G008 - “Just the reassurance, you know, just reassuring the actual person involved…. obviously apologise”, K009 - “I think we should give them a phone call that obviously sorry about that the computer’s…not working properly…you should explain that…obviously you know the people out there, so that they’re not worried or feeling anxious”, M01 - “I would say take responsibility for that, ‘cus that person’s care has been affected…”, G003   Method of communication about errors would depend on the person   - “It would depend on the person, I just…I think as long as you can sort of tell them in some way straight away, whether it’s a phone call, text or letter or…yeah, just I think as long as you can tell someone as soon as possible and just the way you tell them is sort of up for debate really”, M010 - “I guess just once it’s resolved then it should erm, email, text or notifications saying erm ‘this was incorrect’…but I think I don’t think it has to be anything too deep!”, G008 - “I think you’d still have to have a meeting…With the people, everyone concerned”, K007 - “I don’t know, it’s just, like, even a phone call or something to them to, like, look, sorry, the computer made a mistake”, M004 - “Like phoning them up or going to their house or ringing a family member to see if they’re okay or anything like that”, M007 |  |
| 1.3. False positives: blame, unnecessary restriction, and fear of relapse | If alerts are treated uncritically by the clinical team, false positives would be more problematic   - “If I wasn’t relapsing they were saying I was relapsing I could get treated unnecessarily, you know I’ve been treated unnecessarily before you know so you know”, E009 - “You could get an over-reaction, and in the context of mental health, an over-reaction can mean unnecessary restriction, and you really want to avoid that”, K001 - “You wouldn’t want, you know, an app giving the kind of indication that there was a big risk or a big danger when there wasn’t”, K001 - “I would feel like I was being blamed or singled out for something that I haven’t done…That definitely dissuade me”, E009. - “You can’t just be told you’re feeling unwell if you’re not”, E003   Self-fulfilling prophecy, fear of relapse   - “Well if it like if it said you were going to relapse when you actually weren’t, you might actually get into your head like oh I’m going to relapse and then you’re expecting it to happen…It’s more likely to happen when you’re thinking about it like that. When you’ve got it on your mind constantly”, M012 - “For me, it would be the over diagnosing, it would think there’s a problem not there cos then I’d start thinking there’s a problem there, again that feedback loop”, S008 - “I think that would be incredibly useful. It would, at the same time, be potentially anxiety provoking, and there’s always a thought, well a self-fulfilling prophecy, so if, on a conscious level you’re told you could be relapsing then somehow that acts at a subconscious level to either exacerbate the relapse or trigger a relapse. I don’t know if the brain works that way to be honest, but it would be a concern”, K001 - “That’s one of the reasons I think they should contact the, the mental health team and then them contact my trusted ones, because seeing my mental health is ok and then have app tells me your mental health’s not ok and then I would, ‘oh…what’s wrong?’ and my mind start racing thinking what am I doing wrong, why is this prompting me this message, and maybe be a mistake. So saying my mental health wasn’t ok, I would like to know through a person”, G005   Useful to be prompted to pay attention to mental health (even if not relapsing)   - “Yeah, I mean, it’s it’s warning…warning is not always right then it’s not a bad thing I don’t think”, G008 - “Erm, I mean it’s, it’s, I don’t think it’s, well it’s not any different cos if it were to tell you you’re becoming more and more unwell and it raised, erm, like this message with your CPN, once you’ve spoke to your CPN and you’ve established that, it your mental health hasn’t been, erm, hasn’t been deteriorating then it’s better to have that there covering youse and it is not being there – if that makes sense?”, G008 - “I think if the first situation that the computer told me that I’m unwell but actually I’m fine, probably I’m just, **will be more alert to my situation** and I’m really fine, the computer is wrong but sometimes it’s good to have the alert even though it’s might be not accurate”, G009 - “I don’t think it’s a problem even if they make mistakes ’cause it could be just a good thing to just check in because there’s something that has shown there is a bit of a problem”, M005 - “I’d like to think if it does bring a concern up and I thought it was wrong, it would at least make me think about it sort of thing, like make me think twice or…I don’t think it’s a bad thing, yeah”, M010 - “I think if, if it got it wrong and said that I was having a relapse I I would be really concerned…But I think I know myself enough that praps after a day or so and I'm still alright…I'd know”, S007 |  |
| 1.4. False negatives: “wasted opportunity”, and “false sense of security” | Missed opportunity to intervene early in the relapse process   - “It’s false negatives that would worry me…‘Cause I really, I want to know if there is going to be a relapse, because then I can do something. If I don’t know, it’s a wasted opportunity…If I know beforehand, I could make a real difference to outcomes and experiences for myself”, K001 - Worse if “it didn’t pick up that you were getting unwell…Because you need help when you’re unwell”, K007 - “I think it would be worse if the computer didn’t pick up anything to say you’re getting unwell…It would be worse for the…person in the long run. They could become very ill, you know, nothing’s done but it could be too late before any action’s taken”, K009 - “I think if it doesn’t get it right, I think I’ll feel like disappointed and upset, because they need the help and support, so I think it’ll be like disappointing really, because if it does say like…if I’ve had a bad day and that’s saying I’ve had a good day, then I’ll be like disappointed and stuff”, M007 - “Yeah but then also if it like doesn’t pick up on a relapse then that could also be like a bad thing just because like you might get too deep into it and not want to reach out anymore”, M012 - Although false positives are worse for them personally, “but grand scheme wise, definitely the other way around, people essentially if people are falling through the gaps it brings up a lot of, it brings up a lot of alarm bells for a lot of people I guess”, S007 - “If they don’t pick up that I’m getting unwell and…I’ll tell people that I’ll ring them or whatever, but I really, really, really won’t. So obviously it’s just one of those, I’d just be stuck on my own more than anything”, M004   False sense of security   - “Providing a false sense of security maybe that would be unhelpful as well?”, E009 - In response to direct question about which would be worse (false positives or false negatives): “both of them because of opposite reasons”, G005 - “…and if you were getting worse, and it tells you’re getting better, which is the opposite direction, you would keep getting worse, thinking you were getting better and then you wouldn’t, it wouldn’t help you in anyway”, G005 - “For me it would be worse if it wasn’t picking up when I was getting unwell…that’s more important for me than it thinking that I’m more unwell than I think I am”, M011 - “The tricky one would be if it said you’re fine and you weren't…because that sort of reinforces what the voices are saying…what they want”, S007   Not doing what it was designed for   - “Erm, probably if it missed a relapse. I think that would be worse…as it’s not really doing the job it was designed for, erm, and I can kind of know when I’m going to relapse”, G007 - “I’d like to think if you’ve filled all these specific questions and gone through a specific process, I’d like to think it would be able to pick up on it before, because the whole point of it is I think to pick up on it before you do, so if you realise a bit before the computer has, it’s almost pointless and…that would eliminate the purpose of it in the first place basically”, M010 |  |
| **2. Human-in-the-loop** | |  |
| 2.1. Human oversight, feedback and responsibility | Incorporate a feedback mechanism   - “[If] the computer got it wrong…my mental health team, after checking with my trusted ones and seeing that I was a fine, say the app said that…I was low, they would be able to, to have the, the sliding thing and they would calibrate that”, G005 - “I think it’s a very practical thing, You’d really need to get it, get it working and do that…Give it some kind of feedback, do it like that”, G002 - “Whether there would be a facility for maybe friends or family of the individual that's part of the team to question whether it’s right or not. Or whether the individual, if they're in the right frame of mind and they're fully conversant as could give feedback…So yeah, I suppose it's they would have to be a teething, period, and make people aware that you know, there could be like a month period where you say you might receive messages that are not always correct. So if you do feel that they're out of sync with what is actually going on with you as an individual give us feedback and let us know”, C005 - “it needs to be some sort of additional questions or more checks for the individual, make sure the computer gets it right because if it’s quite vague and the computer get’s it wrong then there is no way for it to improve, so there needs to be some sort of alternative question…or maybe even some sort of…timeline for the individual to see…how their mental health has been over the weeks so they can just process, oh I was feeling sad that week and wanted to stay in or you know there was nothing necessarily wrong, okay that day was quite bad cos I really didn’t do anything I didn’t leave the house for three days straight, I think there needs to be additional questions”, C004 - “I think as long as I felt I was able to have my view taken into account”, G001 - Another found it reassuring to know who to contact when the system appeared inaccurate: “It would be good to know that I could ring up somebody and say look this is saying that I'm unwell and I don't feel unwell what should I do?”, S007 - “I think you should employ people to monitor that…So whenever the programme makes a discovery or a pattern, that gets notificated to the team that you make, and then that team debates whether that is right or wrong…And then that helps the programme work out things better as it goes along then”, C006   Who should provide human oversight and bear responsibility – clinical team or someone else?   - “I don't think I'd be averse to it as long as I think there were safeguards put in place to say that there is still human oversight over”, C005 - “You’ve got to make sure that someone’s got the job where they look at the patterns and actually figure out whether those are patterns that are actual patterns or not”, C006 - “So you got the human intervention updating and tailoring it. Uh. I suppose every maybe a month or so, just to make sure that it's actually doing what it's supposed to, rather than misunderstanding or reaching conclusions without actually consulting any facts”, C005 - “I think as long as you have a team that are evaluating it every x amount of days, weeks, then I think it’s fine. I think it’s obviously it’s a shame when it happens in the moment but as long as there’s not a hundred per cent certainty that…or a hundred per cent dependency and then there’s other avenues of help still available, I think it’s fine”, M008 - “I think you just maybe have to implement like an oversight committee and whether it was. I think if if it's owned by private means and then it's controlled by a number of governments with invested interest, I think I'd be OK with that. I would be slightly more concerned if it was just you said, information being collected and then analyzed and decisions being made by private individuals, because you know, most CEOs and companies that own AI facilities are not elected, they're elected by shareholders and like most shareholders, unless you can afford to buy the cost of the share, then you don't really get to have a vote anyway and so as I said, I think if if if you are going to use AI machine learning there would need to be, um greater oversight via either like NGOs or you know have intergovernmental organizations just keeping an eye on it. If it is going to be run by private corporations, yeah, OK, but it's that sort of fine line. And like you say, if it was to go into the sort of private sector and to make sure what what that would look like and comparing it to how it would look in the health service and things like that”, C005 - “there needs to be some human involvement…because…the computer programme might make mistakes that a human would have to, someone would have to answer for”, K007. |  |
| 2.2. Mitigating false positives | ***2.2.1. “We can talk about it”***  False positives less of a concern if relapse alerts trigger contact from the clinical team   - “If it thinks that I’m getting unwell and my mental health team get in contact…I can tell them that I’m fine”, M004 - “Well that’s why it should be supervised by a human. So if it’s, so that if its taking data that’s not making sense, and if its interpreting it wrong then a human I think would be more…to say…the machine’s got it wrong it’s some other way you know”, E008 - “For me to see the information, speak to them on the phone, arrange an appointment face-to-face with them to talk about these things, information gathered and what would be the next step after that”, E003 - “Yeah, you'd just say oh no I'm fine”, to the care team, S003 - “It wouldn't piss me off or anything or like that if it just got it wrong and I was literally alright”, S006 - “If there’s a mistake with the computer then…I’d get in touch with my care coordinator or just talk to my family or you know friends let them know I do feel well I’m not hearing voices. Obviously, I’d explain this to my care coordinator and my family and friends”, M014 - If the algorithm got it wrong: “Em…I just have to ask, I just have to go and see my psychiatrist and argue my case or whatever, I don’t…I don’t know”, E009 - Regarding false positives: “Then you need a bit of human interaction don’t ya”, S002   DRM system should not be making clinical decisions   - “How much of the machine learning makes its own decisions without then having to consult, I suppose someone that is looking at it from an ethical and moral point of view? Yeah. So making sure that that human guidance and supervision also it's not solely just being left to make decisions on its own. So there are people that are checking it and making decisions about the individuals as well”, C005 - “As long as it wasn't making clinical decisions, it was just being used as information”, G001   False positives less of a concern than false negatives because they can be mitigated by talking about them.   - “I’d rather it picked something up and be wrong than not pick something up. Because obviously if it picks it up and it’s wrong, well, you know, we can talk about that, but if it doesn’t pick up but then I do something wrong and the computer didn’t spot it, then it’s not good for the computer, is it?”, S001 - “I would say…a missed diagnosis would be worse erm for me because would prefer someone to say do you need an intervention and I can say I’m fine I’ve just been having a bad few days rather than make no contact at all”, S005   General procedure in response to relapse alerts should be to get more info from the person. These quotes were not directly mentioned in relation to false positives (although some imply that following up app information may mitigate false positives)   - “They maybe should like talk to you…to kinda confirm what the data from the phone says. Yeah, somebody could get through talk to you kinda really quickly but it’s a lot better”, E007 - “I think if the computer suspects there’s something wrong or the technology suspects there is something wrong then you should be seeing the psychiatrist, or the psychiatric nurse to make a further assessment to check you know what I mean? Its only, it’s like a triage, a nurse will decide and hand you onto a doctor”, E008 - “I think it might help them do visits more like if they’re noticing, let’s say me for example, [name] is getting iller over time and we’ve noticed his score is going all the time, maybe we’ll book a visit and we’ll go and see him and find out what’s happening”, C007 - “You know I think they’d phone you straight away. Get a wee telephone call fae your CPN asking you how you’re doing. Would be quite a good way I’d think. And then it’s up to your CPN to assess then if you need to go in and see the psychiatrist or not”, E004 - “they would phone me…And get me seen as quick as they could”, G006 - “Maybe phoning me would be the fastest method, maybe, if they think there is something wrong”, G007 - “I guess just receiving like some sort of text or phone call from my eh CPN, I think openness is quite important to have”, G008 - “Probably contact me and then tells me I am worrying, you know…my…situation, is GP aware? And then go out more…and if, or, and if I’ve already joined some groups that they have..”, G009 - “Either a phone call or coming out to the house”, G010 - “So maybe the app can be a way to notify someone that there’s an issue and then the person will come in and you can have that discussion”, M005 - “By phone obviously contact me by phone maybe send me a message to get in touch with my care co-ordinator obviously or you know chat with me and stuff like that really, see how I’m feeling”, M014 - “Well I would like them to contact me phone me up or something to see how things are”, S003 - “I'd quite like them to check in on me, do you know what I mean. It's like, it would be quite, quite helpful because as I say I struggle reaching out, and I'm sure a lot of other people do as well”, S006 |  |
| 2.2. Mitigating false positives | ***2.2.2. Contextualising passive data***  Examples of decontextualised passively sensed data being misinterpreted by the algorithm   - “If someone doesn’t leave the house and it’s not to do with their mental health, would that skew their result, would that impact it?”, C004 - “For example 2 or 3 times a week I’ll just sleep til like 1pm and I’m not relapsing or anything, I’m just enjoying a little break”, C003 - “Yeah, sometimes computers can misunderstand things, details that is wrongly read, to understand it wrongly, yeah. Like me, yeah, I have children, my house is noisy. Probably, it will pick up noise if it's able to do that, it might say, oh there's a problem here, and there is no problem”, M006 - “I go on my exercise bike once a day it might think my heart's risen”, S003 - “…say but it's telling me that I'm displaying, you know, uh, an extremely high heart rate and that I need to calm down with this going to spike my anxiety when you might have actually just been going for a run everyday. CONNECT failed to recognize that it's a run and it thinks that you're just extremely stressed”, C005   Someone who knows me can use additional context to interpret the algorithm’s output   - “I guess it’s tied to people that know me so they’ll know better if I am not ok…cos erm like for example 2 or 3 times a week I’ll just sleep til like 1pm and I’m not relapsing or anything, I’m just enjoying a little break”, C003 - “I think if it was sent to the care team and then the care team haven't been given the information that the AI is coming to by Machine learning is is you call it they're letting the care team reach its own conclusions as well, stacked on the information that's been gathered because, you know, you might have had someone that's been out to visit them two weeks ago from the care team and said, oh, well actually it’s because they just recently you know, split up with their partner or yeah and is you know working multiple jobs or but in between jobs or you know there's because as you said, life gets in the way and it's not until you get an update of someone um, about what they're doing and what they're what their purposes and was and what their objectives are that you can really understand why their erratic behaviour would be. And you said whether it's out of the normal behaviour? So yeah, I think as long as the AI information is given to the care team, then we will probably have a more personal connection with that individual”, C005   Active Symptom Monitoring can provide context   - “You would be able to argue your case because you’d have wrote it down. So I mean bring the app and you’re saying there’s obviously there’s a problem with the calculation on this app cos look at it because here’s my monthly input here for January, for example, and as you can see I’ve been fine so why is that highlighting that I’m needing help?”, E004 - “you were mentioning about the phone knowing that I’m home, seeing if I’m not texting people that I used to text, it could give, pop up a message, are you alright?…”, Are you enjoying staying at home or you’re staying home for another reason that you…that you’d like to share?”, G005 |  |
| 2.3. Mitigating false negatives | False negatives are not too problematic if the system is only used as a part of someone’s care rather than being fully relied on   - “You can always seek help…if you know yourself. If you don’t know, then it’s a psychiatrist’s job to work it out, it’s not the machine’s job. It couldn’t be used as a replacement for a psychiatrist”, E001. - “If it misses you’re deteriorating then that would be the same if there wasn’t an app there anyway. Erm, so…I think it’s not too bad. Erm, because it’s only offering things that are better than they’re currently there. Like it’s not making anything worse I’d say”, G008 - “And then the other, like computer not even notice that and then, but I think it’s talk my key worker directly bec, because the computer is just an additional thing”, G009 - “It's down to me really isn’t it to reach out, I can't rely on everything to do that for me…yeah I wouldn't, I wouldn't be annoyed or anything like that”, S006 - “Well, you just need to fall back on practical stuff that you did before, take calls on your landline or mobile then from [name]”, G010 - “as long as there’s not…a hundred per cent dependency and then there’s other avenues of help still available, I think it’s fine”, M008 - “It's down to me really isn’t it to reach out, I can't rely on everything to do that for me”, S006 |  |
| **3. Trust, fears and choice** | | |
| 3.1. Human-in-the-loop facilitates trust in algorithms | Notes from analysis diary emphasising the importance of the relationship between the service users and clinician   - “The relationship with the care team is key. It takes a long time to build trust”, analysis diary: G003 - “Participant emphasises trust as a key factor”, analysis diary: E009 - “It's like the app is an extension of the relationship with the team, so the willingness to engage is directly affected by the relationship with the team”, analysis diary: general - “I'm wondering whether there is a general pattern across participants that people with a good relationship with their team are more willing to use an app like this”, analysis diary: G007   Mutual trust   - “I know myself more than anyone else, so they would know my word”, C003 - “I think if your team checked in with you…I trust them…I trust them”, G007 - “Yeah because like, it’s like seeing a new doctor, let’s say you’ve seen a new doctor, do I spill all my information out to this new doctor that I’ve never seen before, if you know what I mean, it’s like would you trust a stranger, you know, when you’re in that early stages [of psychosis] you’re like who is this stranger that I’m going to be exposing my life to, I don’t know if I trust them yet if you know what I mean…Maybe if you could do like the odd, not visit, well, you could do like a phone call or Teams call or visit in person, if you do something like that maybe it might build up their trust… ‘cus this would be like a massive leap for people ennit like oh my gosh I’m going to be spreading data and information about myself, I don’t know what they’ll go into and all that, you know you’ll have that paranoid sense in your head, like I’m alright though I’m like go on you can have all the data you want (laughs) that’s my view”, C007 - “I think it can be trusted just by trusting the persons you know, trusting the persons that’s using it. That’s the only way you can trust it. You have to be able to trust the person”, E003 - “a lot of this stuff is relational, I imagine people might get involved if they feel you are trustworthy”, G001 - “Like erm, I dunno urrr, I think there's a level of trust that I've got between myself and my LP and my, and my psychiatrist. So, that would be one thing like that I would be happy to happy to share it as long as it's with the people that I errr, I've been seeing already”, S006   Mistrust and experience of coercion   - “Yeah I mean just all I’d say is you know its em…you know it’s kind of…being on the same page with your psychiatrist. I mean you know I go and see the psychiatrist and I tell them what I want to, you know how I’m doing and stuff like that and then you know, you know I’m in control of what information’s being passed on and then you know if it was recording information about me and patterns and stuff it was flagging stuff up, once again it could incriminate me you know. You know I think I’ve been on the same page with them and one day they come to me and say ‘look you know we spotted this…this machine learning thing em…you know we’re concerned about this’, what are you talking about you know I don’t know anything about this you know. Like once again I wouldn’t like to incriminate myself you know I wouldn’t like you know think we were on the same page and then one day they turn around to me and say ‘oh we’re concerned about you because of this that or the next thing that we’ve eh…we’ve machine learned’ or whatever it is you know”, E009 |  |
| 3.2. Fear of an overreaction | Fear of medication increase   - “If they felt they needed to then they could…double my medication you know”, E009 - “I like sharing my opinions on the things that are going through my head, but at the same time the repercussion of that is more medication, more dosage”, M002 - “One of the things I learned is you know sometimes it’s best not to give all the information to psychiatrists ‘cos they just jump on you with more medication or something negative you know”, E009 - “wouldn’t want to…incriminate myself so to speak. Not…not in terms of criminality but just in terms of you know like eh giving away information that would cause them to act, you know to do something that I find negative you know. I already find the medication that I’m on negative”, E009 - “I guess that people would check, they wouldn't just assume you need sectioned now or whatever. As long as it wasn't making clinical decisions, it was just being used as information”, G001 - “As soon as you say you’re hearing voices it gives carte blanche to eh inject you with any medications they think’s gonna help”, E009   Fear of hospitalisation/section   - “You could get an over-reaction, and in the context of mental health, an over-reaction can mean unnecessary restriction, and you really want to avoid that”, K001 - “I just don’t want to be sectioned again…it’s really hard to get out when you’re sectioned”, G007 - “I think the only thing that would worry me was, maybe worried about going to hospital I guess. That's probably what my worry would be, because it wasn't the best time to be in hospital. That's probably the only worry I would have really”, C001 - “I think it’s a really good thing. What I’d be afraid is if I wrote these things down ken would I be brought back in against my will you know…That’s what would stop me. But otherwise if folk tell me I would send it to the doctors and the staff team”, E003. - “What I’d be afraid is…would I be brought back in against my will”, C001 - “I guess that people would check, they wouldn't just assume you need sectioned now or whatever. As long as it wasn't making clinical decisions, it was just being used as information”, G001   Two participants (C004 and E009) contrasted feeling surprised and out of control with direct collaborative communication   - If the care team “found this information for your mental health and mention that to the individual I think that would maybe make them pull away erm because the information is getting taken from them and they probably haven’t spoken about it. I think it’s very individual, it depends on the person you are seeing and what kind of health service provider are you working with but yeah I think it’s good in a sense, that they can get the information too because that’s what they’re there for. But they need to speak to the individual and find out what they are struggling with. It needs to be communicated in a way that they both feel safe and it doesn’t feel like the individual or the service is going behind each other’s backs”, C004 - “Yeah it’s still all about the individual and what they want, and their mental health, and what they could give to help them in the moment. They can’t just take charge from it, but they also need to be led by the individual and what feels right for them”, C004 - “I go and see the psychiatrist and I tell them what I want to, how I’m doing and stuff like that. I’m in control of what information’s being passed on and then if it was recording information about me and patterns and stuff it was flagging stuff up, once again it could incriminate me. I think I’ve been on the same page with them and one day they come to me and say ‘look we spotted this…this machine learning thing em…we’re concerned about this’, what are you talking about I don’t know anything about this. Like once again I wouldn’t like to incriminate myself. I wouldn’t like you know think we were on the same page and then one day they turn around to me and say ‘oh we’re concerned about you because of this that or the next thing that we’ve eh…we’ve machine learned’”, E009 - “I think I’ve been getting on ok and they come and say to me ‘oh the staff have seen this or that’ and I thought we were on the same page and all of a sudden they turn around and they say that we’re concerned about your drinking and that means that they’re going to do something about it whether that be cutting….cutting down the amount of money I’m given or putting me in hospital or increasing my medication or something like that”, E009   Fears of overreactions may be mitigated by a conversation   - “That would be a good thing ‘cos I think if I told them how I’m feeling, come to an agreement you know about what would be best for me”, E003 - “As long as someone’s honest if it’s wrong or if it’s right, I think it’s fine”, M010   Fear of over-reaction not exclusive to digital.   - “If my mum phones my CPN and says she thinks that I’m no doing great my CPN phones me straight away. Do you know what I mean? And that’s what we’ve got in place since that last section to avoid something as horrible as that happening again. Do you know what I mean? Because my mum said hindsight was a wonderful thing but she could nae turn back what was said. She got the wrong end of the stick but she had to write a letter to the hospital to explain she’d got the wrong end of the stick before I was getting out. Do you know what I mean? It was awful”, E004 |  |
| 3.3. Fear of an underreaction | Digital monitoring will increase clinicians’ already heavy workload and they might not take relapse alerts seriously   - “…probably massively increasing, like, the workload that like already the clinicians have for taking care of your mental health. There's only so much input that they can have and if they then get sent this every single day and sometimes it's misinformation as well, they're just probably going to be reluctant to even take note of it, if you see what I mean. Like it might come through but they might be like, oh, you know, this programme, it's not worked before, so how can we sort of trust it? So I think trusting…yeah, trusting the actual technology is really important because yeah…and it's probably a while off yet before, you know, it's completely accurate. But yeah”, C002 - “I think it could definitely keep them in the , but I think if it’s like the healthcare providers using it, I find that it could be overwhelming in a sense, of trying to accommodate to these individuals and try and find solutions and it could be just slightly overwhelming, umm, so I don’t know if there is a way to delegate that work from the app but I think people who are using devices to maybe only use it on certain days or maybe have another one of their workers umm on different days so that the workers spread out and so that pressure or overwhelming fears don’t just slip in, so I think it is just when they’re working with individuals and when they’re wearing the device it has to be maybe a set time and in a set place, they don’t wanna take it home and then have the wearable device go off and then they have to come in or something like that, I think it would have to be structured in a way when it’s in work and when it’s appropriate and when it won’t become too overwhelming for all the workers”, C004 - “Does it not make the Doctor a lot busier you know? To work all these things”, E003 - “I’m unsure about how people would perceive it’s authenticness…If people would kind of take that information on the results seriously or still want to do further screening or assessments before proceeding any further”, K003 - “I think it would be really, really practical. But I know it won’t happen, it’s not going to happen at all. It’s like you cannae get a reading to the doctors, you’ve got to phone in and see what they’ve given you on the phone”, G010 - “I don’t think it’d be feasible for lots of people to have this App, because already they’re stretched with what they’re doing and then they’ve got to attend to these messages as well…So, I don’t think they’d be acted on very quickly…by the clinical team…And so, you’ll be left with it for days, thinking about your appointment”, K002. - “So, people that you introduce to the App, you need someone constantly monitoring their calls otherwise it’s going to go pear shaped really…People won’t use it because they’ll think it’s not a good App [if they don’t get a response]”, K002   Won’t scale well   - “As I keep saying, it’s the response…Whether it’s a good response or a bad response, you need responding to…And if you haven’t got that onboard then, you know…And you’re going to be lucky, because you’re just creating a vacuum, you’re creating a small amount of people using this, but in the real world there’ll be thousands using it”, K002 - “I think, you know, because you’re just sampling a small number and seeing whether it works, you’re in a privileged position. So, you have to be realistic when people are doing this as a job it’s a different ballgame altogether…. Do they have the time allocated to be able to do that, is there someone on the phone, or someone looking out for them all the time”, K002   Negative impacts of lack of response:   - Would feel rejection if didn’t get response: “maybe me feeling like somebody should help me out in this, where I’m at at the moment…maybe I should receive a little bit of additional support and not getting that and then feeling that rejection”, M011 - Would find it worrying if they filled in the app but didn’t get a response: “Well, I’ve sent this message and why aren’t I getting a response, it must be serious or, you know, all that kind of thing”, K002 - If didn’t get a response then digital monitoring adds to worries: “It’s all very well to say, oh we’ve got this fantastic programme that we’re going to rollout and, you know, you’re going to be able to talk to us. But then if it takes three weeks to respond, it’s passed so, you know, you’re talking about things…you’re regurgitating things that were worrying three weeks ago. So, as well as the worries you’ve got for that day, you’ve got three weeks’ ago worries reminded of too”, K002 - People would stop using: “I think you’ve really got to work on the response and whether it’s going to be suitable for a large-scale rollout…If you don’t have that in place, people are going to get frustrated, and they won’t use it”, K002 |  |
| 3.4. Making choices about digital monitoring and data sharing | ***3.4.1. Choice about using a relapse prediction algorithm***  Must be a choice to use the DRM system, not mandatory and not a condition of care   - “I think people can make their own minds up what they do…It shouldn’t be compulsory, but if they want to do it, I can’t see why not. I think it’s a good idea”, K009 - “I don’t think it should be mandatory, I think it should be voluntary, and I don’t think it should be tied into whether or not you get care. I would suggest that’s unethical”, K001 - “I think everybody should be put on it to trial it to see if it works for them but I don’t think everybody will use it”, E004 - “it’s an individual choice”, G010 - “I think you just the need to give people time and the space to go with it. You can’t force them to”, K003 - “‘people have a choice so you can do whatever you like, if you don’t like it”, K010 - “I’d prefer it to be a choice, not a condition, to be honest”, M005 - “I disagree with the, sort of…with it gating access to mental health. I think there should be as few limitations as possible to access mental healthcare”, M008 - “It sounds like a human rights violation”, to make it mandatory G001 - Re the idea of it being mandatory “I’d be devastated if that happened. It’s a terrible idea. It’s taking away the person’s independence. It’s treating them like a robot controlled by a computer. It’s, even, it’s taking their identity away and it’s being, to being blackmailed, that if you don’t use this device, then you don’t get treatment. It’s a terrible idea”, M003 - “It’s good as long as it’s not forced onto people. As long as its..its…you participate…I think people might be…well some people might be paranoid about the machine and therefore it may make them feel worse because of the paranoia against the machine”, E008 - “Not everyone has a smartphone and a wearable”, M004 |  |
| 3.4. Making choices about digital monitoring and data sharing | ***3.4.2. Choice about sharing algorithm-generated relapse alerts with the clinical team***  Should be a genuine choice whether to share alerts – don’t pathologise those who choose not to share   - “The program I suppose is harmless. I just, I don’t really want the information relayed back to a professional…it’s not all dependent on a mental illness. Some of it’s just choice I think”, E004 - “I just don’t feel anything should be mandatory in mental health because usually there will be somebody it doesn’t work for, then they will just be labelled with treatment resistance or…not willing to engage…You see that with anti-psychotics already and it would just be crap if this becomes like a new…It is good to have options”, G001   Would not personally want to share alerts automatically with a clinician   - “I wouldn’t do it. Yeah. I don’t feel desperate enough”, K002 - Preferred to show clinicians the data in person: “You could share that with them at a meeting or appointment…because then it’s like a two-way conversation…Yeah, I guess that takes away the automatic side of it, and I don’t know, that could be a downfall as well. Because it’s in some peoples’ cases, if things are getting really bad, then it is best for someone else to get involved, but I think you have to look at it case by case”, K004 - Would prefer DRM data to be checked by someone else before it was sent to the clinical team: “I would need to be very bad indeed before I went for help from the team…I would prefer more tolerance to stuff before information was passed on”, (G001); “….through some kind of filter first, like maybe somebody from the [research] team could maybe check in to see if there is actually a problem”, G001   Would wish to share alerts automatically with a clinician   - “I wouldn’t mind… Mainly because well after coming-, when I got a lot better than I was when I first got diagnosed, I used to avoid talking about my mental health… I basically locked myself away from the situation mentally which wasn’t healthy and I’ve learnt from that now, it’s better to have people help you in those situations”, C006 - In response to whether there is anything they would find unhelpful about the clinical team receiving alerts: “Maybe if…I don’t know, maybe if the app did things, sending notifications without telling me...If my mental health was tipping and it was gonna notify someone, it would be good to know first”, C006 - “If it can tell them what to do, that would be fantastic”, K010 - “For me personally, I can’t speak on behalf, obviously, of other people, but for me personally it wouldn’t bother me because I’m quite open with my mental health team as it is, so it’s just, it wouldn’t really make much difference”, M004 - “I think that would be fine, I trust them so far for the past few years, so, I know they are there to help me so I wouldn’t mind what they know, and they know everything basically anyway”, G007 - “Er yeah, no, I think that’s a good idea, I think erm, especially if there’s some sort of information that’s quite critical then erm if I was to have that conversation with erm like yourself over like Teams or in person, then you would raise that with my keyworker anyway, so. I don’t think it should be different if it was information from an app”, G008 - “I would be okay with that, I would be okay with it…because they share all the information, so it’s okay”, M005 |  |
| 3.4. Making choices about digital monitoring and data sharing | ***3.4.3. Sharing algorithm-generated relapse alerts with family members/carers***  Consent from the person with psychosis about whether to share and who to share with is essential; some would not choose to share with family for specific reasons.   - “If it was with permission then I think it’s completely fine”, M008 - “as long as they give their consent to say yeah, let them know if I'm struggling then carry on”, C005 - “As long as they give consent to do it, like I’d be happy enough for my parents to know where I am, if you know what I mean, like if my health is deteriorating, they do keep an eye on it, I think it’d be good. But it’s the same as we said, like a different angle that I’m looking at it now because I’m tryna see both sides, it might be like oh I’ve had a fall out with my parents, I don’t want them to know any more if you know what I mean, you might not want them to know anymore”, C007 - “If somebody had already consented”, G001 - “As long as it’s all fairly done, you know, I don’t mind. Ok, so as long as you’ve said it’s okay”, G002 - “Ok, so as long as you’ve given the app permission. Yes”, G009 - “So long as…it doesn’t do it without my consent”, K003 - “I don’t mind with my permission”, K007 - “I think that’s a really good idea with the person’s consent”, M011   Nature of relationship with the recipient is important – needs to be someone you trust and are on good terms with   - “I probably found that quite useful, especially if they are in close contact and they're on good terms. I think that's the key term there would be good terms, um because imagine if you didn't have a very supportive family, yeah I suppose you will feel like they're constantly interfering. Intervening when you wish they weren't. Uh. You know you can, I suppose it's the old saying. You can’t choose your family, but you can choose your friends. And just because blood is thicker than water, it doesn't mean that they are your family. Yeah. So yeah, I think as long as there was a consultation with the person you made aware of the family dynamics as well, that would be extremely useful and as long as they give their consent to say yeah, let them know if I'm struggling then carry on”, C005 - “Aye…and even with people that I trust, like say my family for example, or eerm…if my mum could get a message like what’s going on, you know?”, G005 - “I don’t know because I don’t really like my mum knowing things”, M004 - “…some family don’t talk to each other and they don’t care. Like, they would rather have the other one killed, you know. Some families are serious”, K006 - “Definitely never a family member, ‘cus I don’t get on with my family very well but I, my carer definitely…I share most things with her”, M011   Might negatively affect relationship or may upset family or significant other   - Cautioned that would need to consider how the person gets the info: “if they had information about me…which the doctors and nurses had…it would just be like…cheating on me…paranoid in the relationship don’t want that. I mean I’d let my partner know any way you know….it dinnae used to be like that”, E003 - “I don’t think that’d be a good idea because that would put them under some circumstances, stress, maybe. And it coming from an app would probably not work as well, I’d say maybe just to the professionals and then they can make their plan to sort it out and they notify the parents as its happening”; “it could upset them”, C006 - “I’d probably put my partner down for that because I dinnae like to upset my mum unless I really have tae’”, E004 - “it might be quite frightening for her to get a thing saying her husband’s mood’s low…and he’s thinking of…suicide, I think that’d be quite a big thing to send through a text or an app…I don’t know. Cos she was upset even when we talk about stuff that I did in the past…so if she started getting pings about how low my mood is, I don’t know”, G007   Geographical proximity of DRM recipient important   - “Yeah, it's fine, but me, my relatives are not here, you understand, so yeah, they are not here. So, and if there is a possibility of alerting them, I wouldn’t want to put them in such a panic…I wouldn't want to put them in such a panic, because they are not here, they are not seeing me. But if it's a relative that is close by, like, easily just driving, and just see you face to face, and see for themselves, yeah, that’s fine”, M006 - Especially if might be an error: “So, when somebody sees you face to face they can say, oh that must really be an error”, M006   Family would need information on the DRM system’s accuracy too if they were going to be sent alerts   - “Again, as long as they knew how reliable it was”, K001   The individual might need time to process the information before it is sent to a significant other   - “Erm…er…that’s definitely a tricky one. I think I’d want it to go through a person first, like say if it went to the GP and then the GP, the practitioner decided to then contact my partner, I think I’d feel more comfortable with that, erm, def…from my experience with apps as well, erm haven’t been great (laughs), especially ones that I’ve worked on myself, things go wrong all the time, I know that, so… (laughs)…If like, you’ve had like something happen and you write it down in the app and stuff and like it feels a lot more personal, and then it just badings to your partners phone, you’re like…I need the time to process that not just it to shoot off straight to them and then them come in and try to talk to me about it, I needed the time to decompress, I don’t need the…[inaudible, overlap of speech]”, S008   Relapse alerts might spark a conversation, be a bonding point, and increase understanding   - “I think my own partner would probably find it interesting maybe. If somebody had already consented to…might be interesting, maybe could spark a conversation”, G001 - “if she’s got that it could be a good bonding point for us…We are interested in each other’s health, we are interested in each other’s welfare so that would be a big perk”, G010 - “Yeah, yeah I have spoken to my partner on many times to say look please please please just read up on my different conditions and erm she goes nah I don’t want to. And I sometimes, I get frustrated with that because like sometimes I might act in a way that she feels I’m being off, or moody and I really am not, I’m not I’m just trying to deal with lots of things so we always have, I know this sounds really weird after 14 years, but we always have a debrief almost at the end of our days so I’ll finish work, she’ll finish work and we only have 10 minutes each to talk about our day, what went on and then that’s it we don’t talk about work or anything else we then just enjoy the evening, otherwise it can go on throughout the evening having a good winge, erm but I think it would be a good idea you know cos then she would kind of know what’s going on and my day and at different times so I wouldn’t be opposed to that at all”, S005 - “I feel that it’ll be more helpful for the family members so they can look it up what my illness is like and what to do…what the triggers are on my mental health and stuff like that”; “that they know about my illness and that I’m not having to explain it myself if I’ve got something muddled up and stuff like that”, M007   It would be reassuring and comforting to know that someone was receiving relapse alerts   - Next of kin receiving alerts “would make life easier”, K010 - “It would be of an advantage, if an aged person is using it to monitor their own mental health. And if they are connected with their children, it's good, yeah, it's a good one. So, it just depends on people, basically, anyway, and situations, yeah. Yeah, it's good for some, you know, yeah, just to keep an eye on people. And if it's a young child who's doing that, it's also good, and like an adolescent, and all of those things, like if an adolescent goes through those situations, yes. In those kind of scenarios, it's good” - “I think that’s a really good idea again because carers are the ones that probably feel the most pressure of keeping somebody that’s suffering with complex mental health issues safe, I think they’re the ones that have that emotional investment as well, ‘cus I have a carer and I don’t always share everything with her, but if the app alerted her and said, you know, this is what our findings are like right now, then it gives them a chance to maybe speak to somebody or speak to me even about where I’m at, sort of help me access the support that I need in that moment, I think that’s a really good idea with the person’s consent”, M011 - “Yes I’d feel better, I’d feel as if I’m being watched and looked over which is really good obviously because that’s what I need sometimes when I’m falling ill or when I’m hearing voices or full of anxiety so that would be very useful yeah, very useful”, M014 - “Yeah, yeah that makes sense, so there's something that it would maybe be reassuring and comforting to [my girlfriend]”, S006 - “I think that’s really important, that one, especially for single parent households. Because if somebody is unwell the person needs company to help with the child, you know, ’cause you don’t want the children being taken by the social service. So I think that’s really important that someone – like a friend or a carer – is notified of an incident”, M005   Family could provide additional context outside of the app   - “I think they would…they could reach to people that are close to me and talk to them and try to understand if there is something going on because if they, bec…I I believe people under mental health conditions sometimes when they are bad they wouldn’t, they wouldn’t, sometimes they wouldn’t want to contact the mental health team. But I don’t think that’s a good step, you know. So I think contacting people that are close to us if there are any, hopefully there will be. And then see what action needs to be taken, you know”; “And then of course contacting the person, like mys…me, but try to get the context before and see”, G005 - “Yeah, I don’t think that needs to go through to like an app or anything, I think it can be…as long as you made contact kinda thing that’s the main thing”, G008 - “If anything, it will be more appropriate for maybe close family and friends to get a message, if they consented to them knowing or have an app, because…sometimes…things can be flagged that aren’t really that serious but are interpreted seriously, and then you’ve got a situation where you feel like you’re being watched or everything you do is going to cause people to flag a concern, so…I would be hesitant to do that. I think it’s more important that the person understands like what could be going wrong or what’s happening, and then they can give permission to anyone else who they would want to have access to that information and flag anything”, K004 |  |
| 3.4. Making choices about digital monitoring and data sharing | ***3.4.4. Receiving algorithm-generated relapse alerts oneself***  Even correct alerts could cause stress, anxiety, or negative feelings   - “If then, they are relapsing but like not to the extent of needing a hospital admission, but they just need a bit of support for the time being, and then it turned around and said look they’re struggling a lot more than they are…It could like cause unnecessary stress”, M012 - “you’re relapsing, but the computer said you’re worse than you actually are then it could stress someone out and they could end up worse than they was before”, (the participant linked this to the need for nuanced wording and suggestions of what to do to help with relapse), M012 - “Maybe get anxious, maybe I dunno…maybe a bit anxious.”; “Maybe if it was like…no…it would maybe make you get panicky cos I get like that sometimes so…”, E007 (this participants preferred alerts to go to the team first rather than straight to themselves)   Phrasing of the alerts is important – needs to be nuanced enough not cause unnecessary stress and not to trigger fears about unwanted escalation of care   - “Yeah so like say if the app said to me ‘you’re relapsing’ - instead of saying ‘you’re relapsing’ it could say ‘signs of relapse’, like and then tell you some techniques and stuff you could use to like you know, pull yourself out of it”, M012 - “So for example, if someone has like schizophrenia or they've got severe tendency to repetitive psychotic episodes…I wouldn't advise you getting an alert like we all did last weekend on your phone because that would probably set some people off”, C005 [Note: “last weekend”, was 23/04/23 when the UK government emergency alert system was tested] - “If you were constantly notifying someone with anxiety that they've they've behaviour seems a bit erratic, it might set them off. So it's that individual basis or what works for some people might not work for others”, C005 - “I think that the responses would have to be tailored to the conditions that people have”, C005 - “So it's that individual basis or what works for some people might not work for others”, C005   Could send to family or clinicians first so they can judge whether to send to you   - “If it’s saying it and it’s wrong, it could distress you a little bit. But if you just send it to the doctor and then the doctor can decide whether to say anything, because computer could get it wrong and then you’re thinking, shit, have I done this, and then that [could cause 34:44] issues. So I think it would probably be better to send it, like you said, maybe a next-of-kin like your mu m or your dad and the doctor, maybe not you straight away”, S001 - “If it was me, I would be…say I was the patient and [inaudible 35:07] the app, what you could do is say, right, this is the app, if anything goes wrong, it’s going to go to your mum or the doctor, are you okay with that, say yes, leave it at that. If anything goes wrong, let the doctor see it, they’re professionals at the end of the day. If they think that you need speaking to, then speak to you, but don’t…I wouldn’t send it to the patient every time, because like I said, they can get it wrong, or it could just be a minor problem that’s not really a big issue that’s set them off a bit”, S001 - “So what do you think about the idea that a computer programme won’t always get it right? - “That’s one of the reasons I think they should contact the, the mental health team and then them contact my trusted ones, because seeing my mental health is ok and then have app tells me your mental health’s not ok and then I would, oh…what’s wrong, and my mind start racing thinking what am I doing wrong, why is this prompting me this message, and maybe be a mistake. So saying my mental health wasn’t ok, I would like to know through a person. So like, the app identifies it’s not ok, it goes to my mental health team, it goes to my trusted ones, and then saying my family agrees my mental health is not okay: [name] your mental health’s not okay, what can we do to help?, you know, instead of just getting the message from the computer”, G005   Some found the idea of alerts annoying and unnecessary   - “If you go through psychosis, the amount of input you get is a lot. And then to feel like it's still being sort of jammed down your throat would just be quite, yeah, intense I think”, C002 - “I wouldn’t want it…. Because I know if I’m having a rough time…. And how to get over it…I’m more developed than some service users…. So, I can understand things…and just plod on. I wouldn’t like all this interaction on the mobile phone, it would be awful”, K002   Many liked the idea of getting relapse alerts directly, themselves   - “If the app did things, sending notifications without telling me…if my mental health was tipping and it was gonna notify someone, it would be good to know first”, C006 - “I think that’d be the best idea really, if the app tells you that your mental health is suffering its like a wake up call isn’t it really”, C006 - “Yeah like as a- as someone who uses services you might not realise that something went wrong if you know what I mean. So it would be good to have a like oh hold on you’ve gotta stop doing that like alerts sort of thing”, C007 - “At least it would alert me to the fact that there was something wrong and I needed to fix it”, E004 - “That could work because with my illness over the past 40 odd years, I have insight into a lot of the time to what’s happening. If a machine was to tell me things are not going right and they’re not going right for these reasons and it might just be the nudge I need to go get help or to help myself a bit more than maybe I was doing”, E008 - “I suppose it might be the case that it is maybe picking up on things you have maybe not noticed are tricky as well. It might make you see things in a new light potentially”, G001 - “It’s just that’s going down and down and then the next minute you know it you’re on your knees”, G006 - “The only reason I think that’d be fine is because I was driving about, a couple of years ago I was driving about everywhere, erm, and my doctor kept phoning me and she kept telling me “go to hospital, go to hospital”, erm, there’s a space for you and all that kind of thing then I eventually went. So I wasn’t listening to anyone at the time, other than my head, I knew what was going on around me, but, if there was something saying maybe you should speak to your doctor about going back in hospital it’d be handy”, G007 - “Like if you meet that threshold then erm sometimes, sometimes you’re the last one to be aware of your mental health deteriorating, so, and having that nudge, erm, would be quite useful, but I do think that I’d have to, you should be notified the same time as like your CPN or your, er, next of kin or whatever”, G008 - “It could provide, well to be forewarned, is to be forearmed. So any indication that you’re experiencing some time of relapse, it allows you then to make a proactive response”, K001 - “I think I would welcome it rather than be in avoidance or un-…not noticing it, I think it would be better to receive that information and warnings to…to keeping myself aware of my behaviour and mood patterns. Rather than like just, not know and keep it, or let it go unchecked, yeah”, K003 - “Yeah, that’s better. That’s better, instead of taking it to someone else, yes”, M006 - “I think that would be probably one of the most useful things, I think, being able to have that assessment and it just gives you the chance to introspect a bit and assess whether you think it’s right or maybe you’ve been ignoring something, thinking it wasn’t any early sign and this might give you the, sort of, kick to think that it is and pay a bit more attention to your own health”, M008 - “In a way it wold be umm it'd be a bit of a relief erm because when you live on your own you don't notice things. In a way it wold be umm it'd be a bit of a relief erm because when you live on your own you don't notice things”, S007   Once it alerts you, it could give targeted suggestions of next steps   - “I think that the individual can do that already, but yeah, it would. It would also clarify to the individual at the same time that you are struggling and yeah, picked up on it. But it would be if they wanted to do something about it. It's good that the app can recognise your signs. But it helps the individual's choice if they want to get better and you know it depends again what mental health illnesses they struggle with, I think with depression I definitely, I find that even face to face, even if someone was saying you need to do this, you need to eat properly and such and such is not until you actually want to take care of yourself and that’s when the change comes. And I think it needs to have sort of what can the app do to help now, now that we recognise the signs? Can you do something like self-care, like do you want to try and brush your teeth, do you want to try and eat something today? Something that leads on from the app saying that we recognise your mental health is struggling, because it’s good that it states that but it needs to make someone confident in the sense that they can do something”, C004 - “And your app could be that specific it could tell you what was wrong. By the way [name] your app showing that you’re doing too much of this or you’re doing too much of that or you’ve lost your motivation - what’s causing it?”, E004 - “There could pop, or pop, you know open and it would take me to the app and it would have like, your mental health is…your mental health is a bit low, so these are things you could do to increase it, or people you can contact or steps you would take…you know”, G005   Preferences for delivery format varied across the sample – personal choice important   - “I think that varies from person to person. So, I think they could, erm, at the start maybe sign up for how they want notifications to come through, like I think you do with most apps, erm cos some people check their emails, and some people never check their phones or texts, so I, I probably want it to come through as erm a notification probably, but I mean anything works for me really”, G008 - Some liked in app notifications but some preferred “an email or a text because I find it quite easy to ignore the apps”, M001 - “I think it would be good if everything was in the app. And if there’s no internet or something like that, how would they…maybe a ’phone call?”, M005 |  |
| **4. Benefits of sharing DRM alerts** | | |
| 4.1. Early intervention | Spot relapse early, make changes and get help before things get worse   - “It would give me an indication that I need to look after myself better, or do things which I know help in the context of a relapse, or seek help…That kind of thing would be very helpful”, K001 - “Well if someone’s mental health is getting worse…if they can get the help they need that’s good”, C006 - “A way to stop me from going under”, M001 - “You don't wait for things to get worse”, M006 - “Its proactive and it will help in the short-term and it will also help in the long-term of monitoring people’s mental health states and the progress they’ve made and look out for warning signs of a relapse”, E002 |  |
| 4.2. Triaging according to need | DRM alerts as a triage   - “It’s like a triage”, E008 - “That could be really useful…. just a way to manage the mental health, that they’re trying to manage. They’re trying to manage it with mental health care team that comes out to visit ye and your CPNs and stuff like that but I think it would take them a lot of time. Do you know what I mean? If they knew everybody was on that and they’d just be getting wee texts or wee phone calls as wee alerts ‘you better phone [name] today’, or ‘you better phone Pam down the road today’…so it would help them keep on top of their workload better”, E004 - “If they could work alongside the wards, let’s say I had my psychotic episode, they might be able to say ‘look this is a priority we need to get this person in now’ as well, maybe they’d be able to prioritise who needs it more and who needs it less kind of 'cus I know they always struggle with beds and stuff, wards don’t they, getting people in. I think it would benefit the health board in the long run”, C007   Get information to clinicians quickly; more of a direct line to get help   - “The relevant information being passed on in a timely manner…would be a great advantage”, K001 - “Cus you can’t always get in contact with your GP…usually you’re like seventeenth in the queue…it’s not always easy to, to even get a GP to have a conversation with you when, by the time you try again, you might not try again…so yeah I think this is really helpful”, M011 |  |
| 4.3. Minimising human bias | Reduces human bias   - “I think it’s a really good idea. I think there's a…I think eliminating human bias is always a good option”, M008 - “I guess, like it’s, it’s mitigating that human error that you could get if its done by a person, so, erm I’m kind of all for things like AI or machine learning. So, erm, I think it’s a good idea”, G008 - “I think it’s also then more accurate. Well, it’s more accurate of the information it can gather because if someone’s asking you, do you think like your heart rate is elevated or lowered more than usual and there is no interpretation, it’s just automatic machine gathered information, whereas if you’re going to input it yourself you might be wrong, you might be exaggerating or something like that”, M008   Redistributing power through direct communication   - “I thinks [the DRM with relapse prediction algorithm is] fantastic…Modern psychiatry and psychology still a lot of it is based on old school rules…The nurses are trained to put the fear of god in people to control them right?…I’m absolutely delighted that this has come up at this point in time and…I for one would definitely use it and recommend it to other people that they use it as well. Because a lot of stuff gets…em what’s the word…there’s miscommunication between the staff so sometimes the doctor will say something and it’s not true and I’m like ‘that’s not true’ but they’re like ‘Well I’m the doctor and I’ve been told this’ and I’m like ‘well you know you’ve been misinformed’…There’s an awful lot of people saying one thing and you know the doctor can say anything, they’re all powerful, the power goes to their head. I think this way is much more kinda wholesome even though its advanced technology, because its actually coming from you and there’s not a middle man whose you know for whatever motive they’ve got sticking an oar in it…Eliminating human error and miscommunication is clearly the way forward here”, E002 |  |
| 4.4. Efficiency | Quicker analysis, reducing human workload and freeing up staff for other tasks   - “I think that would great because like it take, would take a long time to process this much of data, and then through machine learning it would be much quicker”, G009 - “Yes, I think you’re also cutting down on like worked hours for human people to like actually have to go and be trained and then assess people and then tabulate all that and perform like a study just to, sort of, do like a meta-analysis of the whole thing”, M008 - “Obviously there’s staff shortages within NHS, if we can like save the staff from having to do a job like that and frees them up for other jobs as well”, M010 - “Yeah I mean if it’s just algorithms that get put together on a, on a computer and it picks stuff up…yeah I think 'cus obviously at the moment in time is, and like the number of people are leaving the health service especially in care they’ve just not got the number of staff for the numbers of patients that they have…. So this kind of technology would be, I think it would be a game changer”, M011.   A responsibility to use the most up-to-date methods   - “If the information was there and if the process had been done, I think it’s, sort of, the responsibility to use the most up-to-date methods to assess and treat people. So I think health services should use it as soon as it was verified and everything”, M008 |  |
