# Supplementary material for: Views of People With Psychosis About Algorithm-Based Relapse Prediction and Data Sharing: Qualitative Study
Source: J Med Internet Res. 2026 Apr 10;28:e86753. doi: 10.2196/86753 (PMC13068192; doi:10.2196/86753)
Supplement: Checklist 1 [file jmir-v28-e86753-s002.docx]

### Consolidated criteria for reporting qualitative research (COREQ) checklist

| **No** | **Item** | **Guide questions/description** | **Notes on relevant content** | **Page of manuscript** |
| --- | --- | --- | --- | --- |
|  | **Domain 1: Research team and reflexivity** | |  |  |
| 1. | Interviewer/facilitator | Which author/s conducted the interview or focus group? |  | Supplementary information S3, reflexivity |
| 2. | Credentials | What were the researcher's credentials? *E.g. PhD, MD* | All had a PhD, DClin or MSc | Supplementary information S3 |
| 3. | Occupation | What was their occupation at the time of the study? | Researchers on the CONNECT study | Supplementary information S3 |
| 4. | Gender | Was the researcher male or female? | All female | Supplementary information S3 |
| 5. | Experience and training | What experience or training did the researcher have? | Relevant degrees and CONNECT training | Supplementary information S3 |
| 6. | Relationship established | Was a relationship established prior to study commencement? | Researcher not usually known to the participant | Supplementary information S3 |
| 7. | Participant knowledge of the interviewer | What did the participants know about the researcher? e*.g. personal goals, reasons for doing the research* | Not known aside from info presented in the participant information sheet | Supplementary information S3 |
| 8. | Interviewer characteristics | What characteristics were reported about the interviewer/facilitator? e.g. *Bias, assumptions, reasons and interests in the research topic* | “All researchers who conducted interviews or analysis had some background knowledge of the topic area and may have brought their own assumptions and biases. For example, they may have felt invested in the idea of a DRM system that includes passive sensing being used within mental health care in future.” | Supplementary information S3 |
|  | **Domain 2: study design** | |  |  |
| 9. | Methodological orientation and Theory | What methodological orientation was stated to underpin the study? *e.g. grounded theory, discourse analysis, ethnography, phenomenology, content analysis* | Thematic analysis  Critical realist epistemology | Manuscript pg. 21  Supplementary information S3, epistemological approach |
| 10. | Sampling | How were participants selected? *e.g. purposive, convenience, consecutive, snowball* | Purposive | Manuscript pg. 20 |
| 11. | Method of approach | How were participants approached? e*.g. face-to-face, telephone, mail, email* | Via mental health services | Manuscript pg. 20 |
| 12. | Sample size | How many participants were in the study? | 58 | Manuscript pg. 20 |
| 13. | Non-participation | How many people refused to participate or dropped out? Reasons? | 11 participants, across all sites  Reasons:   - Keen to "move on with life" - Keen to "focus on life" - Unwell - Despondent with healthcare - Not interested in using technology to monitor mental health - Scheduling challenges due to job hunting and other commitments | Supplementary information S3, Eligible individuals declining to participate |
| 14. | Setting of data collection | Where was the data collected? e*.g. home, clinic, workplace* | In-person or via phone/video call, depending on participant preference and risk assessment | Manuscript pg. 21 |
| 15. | Presence of non-participants | Was anyone else present besides the participants and researchers? | Usually not. One participant’s partner was present. | Supplementary information S3, reflexivity |
| 16. | Description of sample | What are the important characteristics of the sample? *e.g. demographic data, date* | Described in results and Supplementary Information S4 | Manuscript pg. 3  Supplementary Information S4 |
| 17. | Interview guide | Were questions, prompts, guides provided by the authors? Was it pilot tested? | Yes, topic guide in supplementary | Manuscript pg. 21  Supplementary information S2, topic guide |
| 18. | Repeat interviews | Were repeat interviews carried out? If yes, how many? | No, one-off | Manuscript pg. 21 |
| 19. | Audio/visual recording | Did the research use audio or visual recording to collect the data? | Audio recorded | Manuscript pg. 21 |
| 20. | Field notes | Were field notes made during and/or after the interview or focus group? | Reflective logs | Manuscript pg. 21 |
| 21. | Duration | What was the duration of the interviews or focus group? | Range 20-78 minutes, median 43 minutes | Manuscript pg. 21 |
| 22. | Data saturation | Was data saturation discussed? | Data saturation was not meet in this study. The sample size was determined *a priori*, ten participants per site to enable geographical heterogeneity. As Braun and Clake (<https://doi.org/10.1080/2159676X.2019.1704846>) argue, data saturation is not relevant to all types of thematic analysis, particularly reflexive thematic analysis, used in the current study. The decision to aim to recruit approximately 10 per site was a pragmatic one: we considered this a feasible number to recruit with the time and resources available. | Manuscript pg. 4 |
| 23. | Transcripts returned | Were transcripts returned to participants for comment and/or correction? | No | Not mentioned because not done |
|  | **Domain 3: analysis and findings** | |  |  |
| 24. | Number of data coders | How many data coders coded the data? | EE, 4 LEAP members, 2 McPin supervisors | Manuscript pg. 21 |
| 25. | Description of the coding tree | Did authors provide a description of the coding tree? |  | Manuscript pg. 21 |
| 26. | Derivation of themes | Were themes identified in advance or derived from the data? | Derived from the data | Manuscript pg. 21 |
| 27. | Software | What software, if applicable, was used to manage the data? | Nvivo | Manuscript pg. 21 |
| 28. | Participant checking | Did participants provide feedback on the findings? | No | Not mentioned because not done |
| 29. | Quotations presented | Were participant quotations presented to illustrate the themes / findings? Was each quotation identified? e*.g. participant number* | Yes | Manuscript pg. 3-17; Supplementary material S5 |
| 30. | Data and findings consistent | Was there consistency between the data presented and the findings? | Yes | Manuscript pg. 3-17 |
| 31. | Clarity of major themes | Were major themes clearly presented in the findings? | Yes | Manuscript pg. 3-17 |
| 32. | Clarity of minor themes | Is there a description of diverse cases or discussion of minor themes? | Yes | Manuscript pg. 3-17 |
